# Hypoxia-Driven Changes in a Human Intestinal Organoid Model and the Protective Effects of Hydrolyzed Whey

**DOI:** 10.3390/nu15020393

**Published:** 2023-01-12

**Authors:** Ilse H. de Lange, Charlotte van Gorp, Kimberly R. I. Massy, Lilian Kessels, Nico Kloosterboer, Ann Bjørnshave, Marie Stampe Ostenfeld, Jan G. M. C. Damoiseaux, Joep P. M. Derikx, Wim G. van Gemert, Tim G. A. M. Wolfs

**Affiliations:** 1 Department of Pediatrics, School of Oncology and Reproduction (GROW), Maastricht University, 6229 ER Maastricht, The Netherlands; 2 Department of Surgery, School for Nutrition, Toxicology and Metabolism (NUTRIM), Maastricht University, 6229 ER Maastricht, The Netherlands; 3 European Surgical Center Aachen-Maastricht, Department of Pediatric Surgery, School for Nutrition, Toxicology and Metabolism (NUTRIM), 6202 AZ Maastricht, The Netherlands; 4 Arla Foods Ingredients Group P/S, Viby J., 8260 Aarhus, Denmark; 5 Central Diagnostic Laboratory, Maastricht University Medical Centre, 6229 HX Maastricht, The Netherlands; 6 Department of Pediatric Surgery, Emma Children’s Hospital, Amsterdam University Medical Center, 1105 AZ Amsterdam, The Netherlands; 7 Department of Biomedical Engineering (BMT), School for Cardiovascular Diseases (CARIM), Maastricht University, 6229 ER Maastricht, The Netherlands

**Keywords:** whey protein, intestinal barrier function, necrotizing enterocolitis, gut organoid, hypoxia

## Abstract

Many whey proteins, peptides and protein-derived amino acids have been suggested to improve gut health through their anti-oxidant, anti-microbial, barrier-protective and immune-modulating effects. Interestingly, although the degree of hydrolysis influences peptide composition and, thereby, biological function, this important aspect is often overlooked. In the current study, we aimed to investigate the effects of whey protein fractions with different degrees of enzymatic hydrolysis on the intestinal epithelium in health and disease with a novel 2D human intestinal organoid (HIO) monolayer model. In addition, we aimed to assess the anti-microbial activity and immune effects of the whey protein fractions. Human intestinal organoids were cultured from adult small intestines, and a model enabling apical administration of nutritional components during hypoxia-induced intestinal inflammation and normoxia (control) in crypt-like and villus-like HIO was established. Subsequently, the potential beneficial effects of whey protein isolate (WPI) and two whey protein hydrolysates with a 27.7% degree of hydrolysis (DH28) and a 50.9% degree of hydrolysis (DH51) were assessed. In addition, possible immune modulatory effects on human peripheral immune cells and anti-microbial activity on four microbial strains of the whey protein fractions were investigated. Exposure to DH28 prevented paracellular barrier loss of crypt-like HIO following hypoxia-induced intestinal inflammation with a concomitant decrease in hypoxia inducible factor 1 alpha (HIF1α) mRNA expression. WPI increased Treg numbers and Treg expression of cluster of differentiation 25 (CD25) and CD69 and reduced CD4+ T cell proliferation, whereas no anti-microbial effects were observed. The observed biological effects were differentially mediated by diverse whey protein fractions, indicating that (degree of) hydrolysis influences their biological effects. Moreover, these new insights may provide opportunities to improve immune tolerance and promote intestinal health.

## 1. Introduction

Whey is an important co-product of the dairy industry that has long been viewed as refuge [1]. However, over the years, whey proteins and peptides have increasingly been regarded as useful compounds with considerable nutritional value and beneficial biological functions [1,2]. Major whey proteins in cow milk, such as β-lactoglobulin, α-lactoglobulin, glycomacropeptide and lactoferrin, are examples of valuable constituents for human health [1,2,3,4]. Whey proteins are added to infant formula to mimic the higher whey-to-casein protein ratio of ~90:10% in human colostrum and ~60:40% in mature human milk [4] since breast milk, and the protein composition herein is considered the gold standard for infant feeding. Moreover, specific whey proteins such as α-lactoglobulin are used in infant formulas [4] because of their high content of essential amino acids and, after digestion, the occurrence of biologically active peptides [3]. Extensively hydrolyzed and free amino acid formulas are given to infants with cow milk allergies [5]. Although supportive evidence remains lacking, (partially) hydrolyzed formulas are used in atopic infants to prevent allergy or in preterm infants for their perceived benefits in the reduction of feeding intolerance and necrotizing enterocolitis (NEC) incidence [6,7].

In the adult context, whey proteins or their hydrolysates may improve muscle protein synthesis [8,9] and muscle recovery following resistance training [10], may aid in preventing sarcopenia in the elderly [11] and could improve metabolic health [12,13]. Moreover, many whey proteins, peptides and whey protein-derived amino acids have been suggested to improve gut health, for instance, through their anti-inflammatory, anti-oxidant, anti-microbial, barrier-protective and immune-modulating effects [14,15,16,17,18]. Importantly, in some studies of experimental colitis models, high amounts of dietary free amino acids were associated with increased intestinal inflammation [19,20], suggesting (too) high amounts of free amino acids could be harmful.

Collectively, these findings make whey protein and well-balanced (partial) whey protein hydrolysates interesting candidates for the prevention or treatment of several intestinal diseases that have a considerable impact on human health across ages and are characterized by inflammation, intestinal barrier loss and oxidative stress, such as inflammatory bowel disease (IBD) [14,21,22,23], intestinal ischemia-reperfusion injury (IRI) [24] and NEC [15,25].

The diverse beneficial effects of whey proteins can be attributed to the easy absorption of whey protein [26], the easy digestion and postulated faster absorption of whey protein hydrolysates [27,28], the high content of essential and branched-chain amino acids in whey [29] and to beneficial effects of various whey protein derived peptides [29,30]. Many whey peptides, which are derived from whey proteins following hydrolysis by commercial or gastrointestinal enzymes, are regarded as bioactive and are described to display a broad range of biological effects, such as anti-oxidant and anti-microbial activity [29]. Bioactive whey protein-derived peptides vary in size and weight, ranging from several to >15 amino acids and from ~300 Da to ~1500 Da [29]. Interestingly, although, together with the protein source, the degree and technique of hydrolysis determine peptide content and could thereby alter biological function [31,32,33], the aspect of degree of hydrolysis (DH) is often overlooked when beneficial effects of whey proteins and peptides are assessed.

In the current study, we aimed to perform a comprehensive screening of whey protein isolate (WPI) and two whey protein hydrolysates with 27.7% degree of hydrolysis (DH28) and 50.9% degree of hydrolysis (DH51) to investigate their effect on the intestinal epithelium in health and disease. To this end, a novel 2D human intestinal organoid (HIO) monolayer model that enabled apical exposure of the human intestinal epithelium to the different whey protein fractions was adapted to allow for testing of nutritional interventions in a healthy setting and during hypoxia-mediated intestinal inflammation and was used for screening of the effect of the whey protein fractions. In addition, the effect of the different whey protein fractions on peripheral immune cells on two well-described pathogenic strains [34,35,36] (i.e., *Escherichia coli* and *Staphylococcus aureus)* and two probiotic strains (i.e., *Lactobacillus rhamnosus* and *Bifidobacterium longum*) [37,38] relevant for human (gut) health was investigated.

## 2. Materials and Methods

### 2.1. Human Tissues and Ethics

Healthy small intestinal tissue samples were collected from patients undergoing pancreaticoduodenectomy at Maastricht University Medical Centre. The study has been approved by the Medical Ethical Committee (METC 151139 (two donors); METC 134107 (one donor); METC 2019-0977 (two donors)) and written informed consent was obtained. Five HIO lines, derived from small intestines of donors ranging from 50–82 years, 3 males and 2 females, were used for this study, of which four were used for model development, and three were used for whey fraction comparison.

### 2.2. Small Intestinal Crypt Isolation

The protocol for small intestinal crypt isolation and subsequent formation of HIOs was adapted from Sato et al. [39]. After collection from the operating theater, small intestinal tissue was kept in AdDF+++ medium ((Advanced DMEM/F12 (12634028, Thermo Fisher Scientific, Waltham, MA, USA)) supplemented with 10 mM HEPES buffer solution (15630056, Thermo Fisher Scientific), 1× GlutaMAX supplement (35050038, Thermo Fisher Scientific) and 1× Antibiotic Antimycotic solution (A5955, Sigma-Aldrich, St. Louis, MO, USA)) at 4 °C for a maximum of 5 h. Subsequently, tissue was washed, muscle layer was stripped off, and tissue was cut into fragments of 4–5 mm. Tissue fragments were washed with ice-cold chelation buffer (5.6 mM Na_2_HPO_4_ (7558-79-4, Merck Millipore, Burlington, MA, USA)); 8 mM KH_2_PO_4_ (7778-77-0, Merck Millipore); 96 mM NaCl (7647-14-5, Merck Millipore); 1.6 mM KCl (7447-40-7, Merck Millipore); 44 mM sucrose (57-50-1, Merck Millipore); 54.8 mM D-sorbitol (S1876, Sigma-Aldrich); 80 ug/mL DL-dithiotreitol (D0632, Sigma-Aldrich) and incubated in 0.5 M EDTA (46-034-Cl, Corning Incorporated, Corning, NY, USA) in chelation buffer for 90 min on a rotating platform at 4 °C. EDTA was removed and tissue fragments were firmly resuspended in the ice cold chelation buffer with a 10 mL pipette. After settling down of tissue fragments, supernatant was removed and kept on ice. This procedure was repeated ~10 times until the supernatant was clear. The different stored fractions were evaluated with light microscopy, and fractions with isolated crypts were put through a 100 μm strainer (43-50100-51, pluriSelect Life Science, Leipzig, Germany) and pooled in a 50 mL tube with 10% fetal bovine serum (FBS) (F7524, Sigma-Aldrich). The combined fractions were spun down at 300 g for 5 min at 4 °C. The pellet with isolated crypts was then suspended in cultrex reduced growth factor Basement Membrane Extract type 2 (BME) (3533-010-02, Bio-Techne, Minneapolis, MIN, USA) and plated in 40 µL drops on a pre-heated 24-well plate (662160, Greiner Bio-One, Kremsmünster, Austria). After solidification of the BME drops for 30 min, normal HIO growth medium (GM) was added, 500 µL per well, and HIOs were cultured in a 37 °C incubator (21% O_2_, 5% CO_2_).

### 2.3. HIO Maintenance

GM was replaced every 3–7 days, and HIOs were passaged ~1:5 every 7–14 days. For passaging, GM was removed and replaced by ice-cold PBS (10010023, Thermo Fisher Scientific). BME drops were mechanically disrupted by scratching with a pipette tip, and the BME dissolved in ice-cold phosphate buffered saline (PBS) was collected in a 15 mL tube. HIO was then spun down at 400 g for 5 min at 4 °C, and supernatant was removed. The pellet was dissolved in 2 mL 1× TrypLE Express Enzyme (12605010, Thermo Fisher Scientific) supplemented with 10 µM Y-27632 dihydrochloride rho-k-inhibitor (HY-10583, Bio-Connect, Huissen, The Netherlands) and incubated for 1.5 min at 37 °C. Thereafter, HIO where mechanically disrupted with a narrowed Pasteur’s pipette (612-1799, VWR International, Radnor, PA, USA). Dissociated HIOs were washed with 10 mL AdDF+++, spun down at 400 G for 5 min at 4 °C, dissolved in fresh BME, and added to a new 24-well plate. For long-term storage, HIOs were frozen in ice-cold Recovery Cell Culture Freezing Medium (12648010, Thermo Fisher Scientific) and kept in liquid nitrogen. All experiments were performed with HIOs recovered from liquid nitrogen. HIOs from passage numbers 3–15 were used.

### 2.4. HIO Culture Medium

GM was used for HIO maintenance and studying HIOs in a crypt-like phenotypical state. GM consisted of AdDF+++ (Advanced DMEM/F12 (12634028, Thermo Fisher Scientific)) supplemented with 10 mM HEPES buffer solution (15630056, Thermo Fisher Scientific), 1× GlutaMAX supplement (35050038, Thermo Fisher Scientific) and 1× Antibiotic Antimycotic Solution (A5955, Sigma-Aldrich), supplemented with 50% *v*/*v* Wnt3a-conditioned medium, 20% *v*/*v* Rspondin-1-conditioned medium, 10% *v*/*v* Noggin-conditioned medium, 1× B27 supplement (17504044, Thermo Fisher Scientific), 1× N2 supplement (17502001, Thermo Fisher Scientific), 1.25 mM N-acetyl-cysteine (NAC) (A9165, Sigma-Aldrich), 50 ng/mL recombinant human epidermal growth factor (EGF) (AF-100-15, Peprotech, Rocky Hill, JI, USA), 10 nM (Leu15)-Gastrin (G9145, Sigma Aldrich), 10 mM nicotinamide (N5535, Sigma-Aldrich), 500 nM A83-01 (2939, Tocris Bioscience, Bristol, UK), 10 μM SB202190 (S7067, Sigma-Aldrich), 10 µM Y-27632 dihydrochloride rho-k-inhibitor (HY-10583, Bio-Connect) and 1% Antibiotic Antimycotic Solution (A5955, Sigma-Aldrich) (end concentration including Antibiotic Antimycotic Solution in AdDF+++). Wnt3a-conditioned medium was in-house made with L cells stably transfected with pCDNA3.1Zeo- mouse Wnt3a, the basic medium was DMEM (31966, Thermo Fisher Scientific) supplemented with 10% FBS (F7524, Sigma-Aldrich) and 1% penicillin-streptomycin (15140122, Thermo Fisher Scientific), (end concentration 100 U/mL penicillin and 100 μg/mL streptomycin). Rspondin1-conditioned medium was in-house made with HEK-293 cells stably transfected with a plasmid carrying the gene for mouse Rspondin1; the basic medium was AdDF+++. Noggin-conditioned medium was in-house made with HEK-293 cells stably transfected with a plasmid carrying the gene for mouse Noggin; the basic medium was AdDF+++. Differentiation medium (DM) was used to induce differentiation in the HIOs and generate a villus-like phenotype and was adapted from the composition described by Van Dussen et al. and Kozuka et al. [40,41]. In differentiation medium, A83-01, SB202190, NAC and Nicotinamide were omitted. In addition, the concentrations of Wnt3a, Rspondin and Noggin-conditioned medium were decreased to 5% *v*/*v*, 2% *v*/*v* and 1% *v*/*v*, respectively. Besides, the γ-secretase inhibitor DAPT 10 μM (D5942, Sigma-Aldrich) was added to the differentiation medium for inhibition of Notch signaling.

### 2.5. HIO Monolayer Culture

To increase accessibility of the apical side of the HIO epithelial cells, a monolayer culture model was used, in which the apical side of the enterocyte orients upwards [40]. Single cells used for monolayer seeding were derived from full-grown 3D cultured HIOs. Briefly, GM was removed, and ice-cold PBS was used to harvest the HIOs in BME. HIOs were then spun down at 400× *g* for 5 min at 4 °C, and supernatant was removed. The pellet was dissolved in 1× TrypLE Express Enzyme (12605010, Thermo Fisher Scientific) supplemented with 10 µM Y-27632 dihydrochloride rho-k-inhibitor (HY-10583, Bio-Connect) and incubated for 9 min at 37 °C. Thereafter, HIO where mechanically disrupted with a narrowed Pasteur’s pipette (612-1799, VWR International). Dissociated HIOs were washed with 10 mL AdDF+++, spun down at 400 G for 5 min at 4 °C, dissolved in GM, and filtered with a 40 μm strainer (43-50040-51, pluriSelect Life Science) to remove large HIO fragments. The cell suspension was added to a 96-well plate (0030 730.119, Eppendorf, Hamburg, Germany) (RNA isolation HIO experiments) or to a μ-Slide (81506, Ibidi, Fitchburg, WI, USA) that was pre-coated with a 1% BME in PBS solution for at least 1 h, 100 μL per well (96-well plate) or 40 μL per well (μ-Slide). For monolayers in a 96-well plate, another 200 μL GM was added following adhesion of the cells, giving a total volume of 300 μL GM per well. Medium was refreshed every 1–3 days. Apical-basolateral orientation of the enterocytes with the apical side upwards was confirmed by immunofluorescent detection of zonula occludens 1 (ZO1) above the cell nucleus (z-stack) (Supplementary Figure S1).

### 2.6. Immunofluorescence Staining of HIO Monolayers

Monolayers for immunofluorescent (IF) staining of Ki67, cleaved caspase 3 (CC3) and ZO1 were cultured in a μ-Slide (81506, Ibidi). After finishing the experiment, medium was removed, and HIO monolayers were fixed with 4% paraformaldehyde for 20 min. Following fixation, monolayers were washed with PBS, and cells were permeabilized by incubation in 0.1% Triton-X in 1% bovine serum albumin (BSA)/PBS for 20 min. Thereafter, monolayers were washed, and non-specific binding was blocked by incubation with 5% BSA/PBS (CC3) or 10% normal goat serum (NGS)/PBS (ZO1). For IF staining of Ki67, no block step was performed. Monolayers were incubated with the primary antibody of interest overnight at 4 °C. The following primary antibodies were used: polyclonal rabbit anti-Ki67 (ab15580, Abcam, Cambridge, UK), polyclonal rabbit anti-CC3 (ASP 175, #9661 Cell Signaling Technology, Danvers, MA, USA) and polyclonal rabbit anti-ZO1 (61-7300, Thermo Fisher Scientific). Monolayers were again washed with PBS and incubated with the secondary antibody, polyclonal donkey anti-rabbit Alexa 488 (A-21206, Thermo Fisher Scientific), for 1 h at room temperature. Thereafter, monolayers were washed again with PBS and incubated with DAPI (200 μg/mL) (D9542, Sigma Aldrich) for 5 min. After a final washing step with PBS and RiOs water, fluorescence mounting medium (S3023, Agilent, Santa Clara, CA, USA) was added to the μ-Slide wells to preserve an optimal fluorescent signal. Slides were imaged within 2 days after staining with a LEICA DMI 4000 confocal microscope (Leica Microsystems, Wetzlar, Germany). Ki67 immunofluorescence was expressed as the percentage of cells that were positively stained for Ki67. Total number of cells (determined by amount of DAPI positive nuclei) and cells positively stained for Ki67 were counted semi-automatically with QuPath quantitative pathology and bioimage analysis software version 0.20 (University of Edinburgh, Edinburgh, UK) [42]. CC3 immunoreactivity was expressed as the surface area was positively stained for CC3. Total surface area (determined by DAPI positive nuclei surface area) and the area positively stained for CC3 were calculated with Image J software (version 1.51s, National Institutes of Health, Bethesda, AR, USA).

### 2.7. Measurement of Paracellular Barrier Function of 3D HIO

Paracellular barrier function of 3D HIOs was assessed with a method adapted from Xu et al. [43]. To this end, 3D HIOs were seeded in 10 μL BME drops in a μ-Slide (81506, Ibidi), and 17% *v*/*v* FITC-D4 (FD4, Thermo Fisher Scientific) was added to the experimental medium for 24 h. Translocation of FITC-D4 from the basolateral to the apical side of the 3D HIO was imaged with a LEICA DMI 4000 confocal microscope (Leica Microsystems) and a luminal: basolateral ratio (L:BL ratio) of the fluorescent signal was calculated with Image J software (version 1.51s, National Institutes of Health, Bethesda, AR, USA). For 3D HIO cultured under normoxic conditions, 10 HIOs per group per donor were included. For 3D HIOs cultured under hypoxic conditions, this number was increased to 15 organoids per group per donor because of the higher biological variance.

### 2.8. Human PBMC Isolation and Culture

Human peripheral blood mononuclear cells (PBMCs) from buffy coats from four adult donors derived from Sanquin Blood Bank (NVT0526.01) were washed with PBS and counted. Cells assigned for later flow cytometry analyses were labeled with 3 μM 5,-6 carboxyfluorescein diacetate succini-midyl ester (CFSE) (C34554, Thermo Fisher Scientific) in 0.1% BSA/PBS for 7 min at 37 °C. For T cell activation, anti-Biotin MACSiBead Particles loaded with human antibodies against CD2, CD3 and CD28 were used (T cell Activation/Expansion Kit, 130-091-441, Miltenyi Biotec, Bergisch Gladbach, Germany) according to the manufacturer’s instructions (2.5 × 10^6^ loaded Anti-Biotin MACSiBead Particles per 5 × 10^6^ PBMCs). PBMCs (both T cell-activated and non-T cell-activated) were cultured in TexMACS Medium (130-097-196, Miltenyi Biotec) with a starting density of ~300.000 cells/well in a round bottom 96-well plate (CLS3799, Sigma-Aldrich). PBMCs were cultured in a 37 °C incubator (21% O_2_, 5% CO_2_). After 48 h, culture plates were shaken every 24 h on a shaker plate for 10 min (improved CFSE signal during flow cytometry analysis).

### 2.9. Flow Cytometry Analysis of Human PBMC

Non-activated human PBMCs were stained for detection of CD3, CD4, CD8, CD25, CD69 and CD127 according to the manufacturer’s protocol. Activated human PBMCs were stained for detection of CD3, CD4 and CD8 according to the manufacturer’s protocol. In addition, a live-dead marker (LIVE/DEAD™ Fixable Aqua Dead Cell Stain Kit, for 405 nm excitation, L34965, Thermo Fisher Scientific) and CFSE labeling were incorporated in both panels. The following antibodies were used for non-activated PBMC experiments: monoclonal mouse-anti CD3 V450 (clone UCHT1, 561812, BD Biosciences, Franklin Lakes, NJ, USA), monoclonal mouse anti-CD4 PerCP-Cy^TM^5.5 (clone SK3, 332772, BD Biosciences), monoclonal mouse anti-CD8 APC-H7 (clone SK1, 641400, BD Biosciences), monoclonal mouse anti-CD25 PE-Cy^TM^7 (clone 2A3, 335824, BD Biosciences), monoclonal mouse anti-CD69 PE (clone FN50, 555531, BD Biosciences) and monoclonal mouse anti-CD127 APC (clone eBioRDR5, 17-1278-42, Thermo Fisher). Antibodies used for activated PBMC experiments were: monoclonal mouse anti-CD3 APC-H7 (clone SK7, 641415, BD Biosciences), monoclonal mouse anti-CD4 PerCP-Cy^TM^5.5 (clone SK3, 332772, BD Biosciences) and monoclonal mouse-anti CD8 PE-Cy^TM^7, (clone SK1, 335822, BD Biosciences). Stained cells were acquired on a FACS Canto II (BD Biosciences) flow cytometer equipped with FACS Diva software (version 6.1.2; BD Biosciences).

For both the unactivated and the T cell-activated experiments, single cells were selected from PBMCs in forward, and side scatter and live cells were selected with a live-dead marker. From this population, the CD3+ cells were gated, and the amount of CD4+ and CD8+ cells was determined. In unactivated PBMCs, the percentage of Treg (CD4+CD25^high^CD127^low^ T cells) was calculated from the population of live CD4+ T cells. In addition, the percentage of Treg that is CD69+ was calculated, and the Mean Fluorescent Intensity (MFI) of CD25 of the total population of Treg was measured. In T cell-activated PBMCs, the percentages of live CD4+ T cells and live CD8+ T cells proliferated were calculated.

### 2.10. RNA Isolation and Quantitative Real-Time PCR

RNA from HIO monolayers and human PBMCs were isolated with the RNeasy Plus Micro kit (74034, Qiagen, Hilden, Germany) according to the manufacturer’s instructions. The RNA concentration of the samples was determined using the NanoDrop™ 1000 Spectrophotometer. Subsequently, cDNA was synthesized with the SensiFAST™ cDNA Synthesis Kit (BIO-65053, Meridian Bioscience, Cincinnati, OH, USA). Quantitative real-time PCR was performed on the LifeCycler^®^ 480 qPCR machine (Roche Molecular Systems, Inc.) with a three-step program for 40 cycles and SensiMix SYBR Hi-ROX Kit (QT605-05, Meridian Bioscience, Cincinnati, OH, USA) was used for cDNA amplification. 5 ng cDNA was used per reaction with a primer concentration of 250 nM (both for forward and reverse primer). Primer sequences were derived from PrimerBank [44].

An overview of the primers that were used is presented in Table 1. RT-qPCR data were analyzed using LinRegPCR software (version 2016.0, Heart Failure Research Center, Amsterdam University Medical Center, Amsterdam, The Netherlands). The geometric mean of the three housekeeping genes (beta-actin, glyceraldehyde 3-phosphate dehydrogenase (GAPDH) and 14-3-3 protein zeta/delta (YWHAZ) for HIO monolayer experiments and CD3 epsilon (CD3e), GAPDH and YWHAZ for PBMC experiments) was used for data normalization.

### 2.11. Microbiology Experiments

The effects of whey protein fractions on the growth of two pathogenic bacteria strains (*Escherichia coli* ATCC 25922, *Staphylococcus aureus* ATCC 29213) and two probiotic bacterial strains (*Lactobacillus rhamnosus* and *Bifidobacterium longum*) was investigated. These strains were plated onto blood-agar plates (BD Biosciences, Franklin Lakes, NJ, USA) and incubated overnight at conditions described in Table 2. A single colony from each culture plate was used to inoculate the appropriate medium (Table 2). Subsequently, cultures were grown overnight in a shaker. Thereafter, saturated bacterial suspensions were diluted in their medium to a concentration of 10^4^–10^5^ CFU/mL, and 100 μL of each suspension was added to 100 μL of a 10-fold serial whey protein fraction dilutions (200 μg/mL to 0.002 μg/mL) in a round-bottom 96-well plate (650185, Greiner Bio-One). Medium without addition of the whey protein fraction was used as a growth control. In addition, as control for growth inhibition, 100 μL of each bacterial suspension was mixed with 100 μL medium supplemented with an antibiotic, as described in Table 2. After incubation, the optical density at 600 nm (OD) of the plates was measured in a Victor^3^ 1420 Multilabel Counter (Perkin-Elmer, Waltham, MA, USA).

### 2.12. Whey Protein Isolate and Whey Protein Hydrolysates

WPI, DH28 and DH51 were provided by Arla Foods Ingredients Group P/S, Viby J., Denmark. For organoid experiments, WPI, DH28 and DH51 were mixed with AdDF+++ at a stock concentration of 6.82 mg/mL (GM experiments) or 1.15 mg/mL (DM experiments). For PBMC experiments, fractions were mixed with TexMACS medium at a stock concentration of 5 mg/mL. Stock solutions were filtered with a 0.22 µM strainer and kept at 4 °C in the dark during the duration of the experiment. For final experiments in organoids and PBMCs, stocks were diluted to a final concentration of 1 mg/mL in the appropriate medium. This concentration was based on earlier studies in which (whey) protein compounds were tested on (human) intestinal epithelial cells [45,46,47,48] and PBMCs [49,50] in vitro. For microbiology experiments, WPI, DH28 and DH51 were diluted in the appropriate medium, and serial dilutions ranging from 200 μg/mL to 0.002 μg/mL were used. Information about the intervention composition is displayed in Table 3 and Supplementary Table S1.

### 2.13. Experimental Set-Up

**Figure 1:**
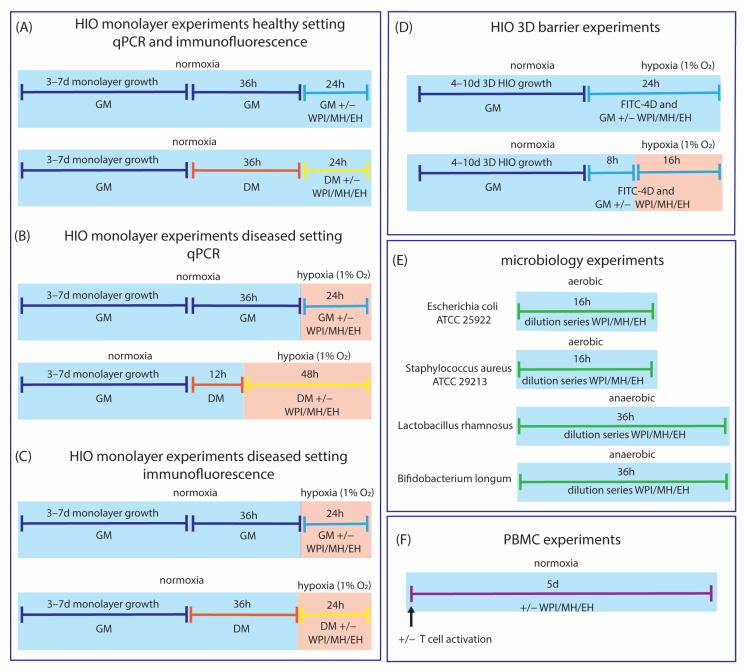
Experimental set-up of the different experiments performed. Light blue blocks indicate physiological culture conditions; light red blocks indicate a diseased (hypoxic) setting. Dark blue lines indicate use of GM; light blue lines indicate use of GM with added WPI, DH28, or DH51; orange lines indicate the use of DM; yellow lines indicate use of DM with added WPI, DH28, or DH51; green lines indicate the use of the appropriate culture medium for microbiology experiments; and purple line indicates the use of the appropriate culture medium for PBMC experiments. (**A**) For HIO monolayer experiments in a healthy setting (normoxia) analyzed by qPCR and immunofluorescence, HIO monolayers were cultured for 3–7 d prior to the onset of the experiment. At start of experiment, crypt-like-organoids were cultured with GM for 36 h, followed by 24 h incubation with GM with or without added WPI, DH28, or DH51. Villus-like organoids were cultured with DM for 36 h and subsequently for 24 h with DM with or without added WPI, DH28, or DH51. (**B**) For HIO monolayer experiments in a diseased setting (hypoxia) analyzed by qPCR, HIO monolayers were cultured for 3–7 d prior to the onset of the experiment. At start of experiment, crypt-like-organoids were cultured with GM for 36 h, followed by 24 h incubation in a hypoxic incubator (1% O_2_) with GM with or without added WPI, DH28, or DH51. Villus-like organoids were cultured with DM for 12 h and subsequently for 48 h in a hypoxic incubator (1% O_2_) with DM with or without added WPI, DH28, or DH51. (**C**) For HIO monolayer experiments in a diseased setting (hypoxia) analyzed by immunofluorescence staining, HIO monolayers were cultured for 3–7 d prior to the onset of the experiment. At start of experiment, crypt-like-organoids were cultured with GM for 36 h, followed by 24 h incubation in a hypoxic incubator (1% O_2_) with GM with or without added WPI, DH28, or DH51. Villus-like organoids were cultured with DM for 36 h and subsequently for 24 h in a hypoxic incubator (1% O_2_) with DM with or without added WPI, DH28, or DH51. (**D**) For paracellular barrier experiments with 3D crypt-like HIO, HIO was cultured for 4–10 d prior to the onset of the experiment. 24 h before analyses, FITC-D4 was added to the GM with or without supplemented WPI, DH28, or DH51. The 3D HIO tested in a normoxic setting remained in the 21% O_2_ incubator until analysis, whereas HIO tested in a hypoxic setting were transferred to a hypoxic incubator (1% O_2_) 16 h prior to analysis. (**E**) The four different strains used in the microbiology experiments were cultured aerobic (*Escherichia coli* and *Staphylococcus aureus)* or anaerobic (*Lactobacillus rhamnosus* and *Bifidobacterium longum)* depending on circumstances physiological to the strain used for 16 h (*Escherichia coli* and *Staphylococcus aureus)* or 36 h (*Lactobacillus rhamnosus* and *Bifidobacterium longum)* in presence or absence of different concentrations of WPI, DH28 or DH51. (**F**) PBMCs were activated or non-activated at start of culture with anti-Biotin MACSiBead Particles loaded with human antibodies against CD2, CD3 and CD28. Thereafter, cells were cultured with or without added WPI, DH28, or DH51 for 5 days before analyses.

An overview of the experimental set-up is displayed in Figure 1. Additional experiments for model development are described in Supplementary Figure S2. Following monolayer seeding, HIO monolayers were cultured with GM for 3–7 days to reach ~80–90% confluence. Thereafter, monolayers were cultured for 36 h with GM or 12 h with DM (healthy setting qPCR/immunofluorescence experiments and diseased setting qPCR experiments). Subsequently, GM/DM with WPI, DH28, or DH51 was added to the cells. GM/DM without adding WPI, DH28, or DH51 was used as control. To induce hypoxia- mediated intestinal inflammation, monolayers were placed in a hypoxic incubator (1% O_2_, 5% CO_2_ and 37 °C) for 24 h (GM) or 48 h (DM qPCR experiments). A longer hypoxia period was chosen for DM-cultured HIO monolayers than for GM-cultured HIO monolayers, as DM-cultured HIO were observed to be less sensitive to the effects of hypoxia (unpublished observations). For immunofluorescence monolayer experiments, the hypoxic period was shortened to 24 h (and the normoxic pre-culture with DM prolonged from 12 h to 36 h) as too many cells were lost during the washing steps of the immunofluorescence staining following 48 h hypoxia. After hypoxia, HIO monolayers were directly processed for RNA isolation or fixed for immunofluorescence staining to prevent a longer period of re-oxygenation. A 3D HIO for paracellular barrier experiments was grown for 4–10 d before the start of the experiment. GM with FITC-D4 and WPI, DH28, or DH51 was added 24 h before imaging. GM with FITC-D4 but without added WPI, DH28 or DH51 served as control. To induce barrier loss, 3D HIOs were placed in the hypoxic incubator (1% O_2_, 5% CO_2_ and 37 °C) for 16 h. HIOs were imaged within 2 h of removal from the hypoxic incubator to minimize the effects of re-oxygenation. For barrier experiments, a hypoxia time of 16 h was chosen, instead of 24 h in other experiments, as this gave a steady loss of barrier function in the hypoxic organoids while retaining a good barrier function in the normoxic controls, and this allowed for pre-incubation of the whey protein fractions before start of hypoxia within a total experimental duration of 24 h. Microbiology experiments were conducted for 16 h (*Escherichia coli* and *Streptococcus aureus*) or 36 h (*Lactobacillus rhamnosusus* and *Bifidobacterium longum*) under circumstances physiological to the studied strain (aerobic for *Escherichia coli* and *Streptococcus aureus*; anaerobic for *Lactobacillus rhamnosusus* and *Bifidobacterium longum*). PBMCs were cultured for 5 days in the absence of WPI, DH28 and DH51 before flow cytometry analyses and RNA isolation.

### 2.14. Statistical Analyses

We performed statistical analyses with GraphPad Prism (Version 9, GraphPad Software Inc., La Jolla, CA, USA). Data are presented as median with interquartile range for all read-outs except for microbiology experiment data, which is presented as mean and standard deviation. For qPCR results, delta CT values were used for statistical analyses. qPCR data are presented as fold change compared to the control group (calculated from delta-delta CT values by 2^−ΔΔCT^). Differences between groups for experiments regarding HIO model development were analyzed with a Mann–Whitney U test (two groups compared) or a Kruskal–Wallis followed by Dunn’s post hoc test (more than two groups compared). Experiments with a comparison of the different whey fractions (both HIO monolayer experiments and PBMC experiments) were analyzed with a Friedman test followed by Dunn’s post hoc test. Barrier experiments with the different whey fractions were analyzed with a Kruskal–Wallis followed by Dunn’s post hoc test. Last, data from microbiology experiments were analyzed with a two-way ANOVA with a Tukey post hoc test. Differences are considered statistically significant at *p* ≤ 0.05. Differences with a *p*-value > 0.05 and ≤0.10 are reported as trends.

## 3. Results

### 3.1. Model Development

#### 3.1.1. Differentiation from Crypt-Like to Villus-like HIO Monolayers

**Figure 2:**
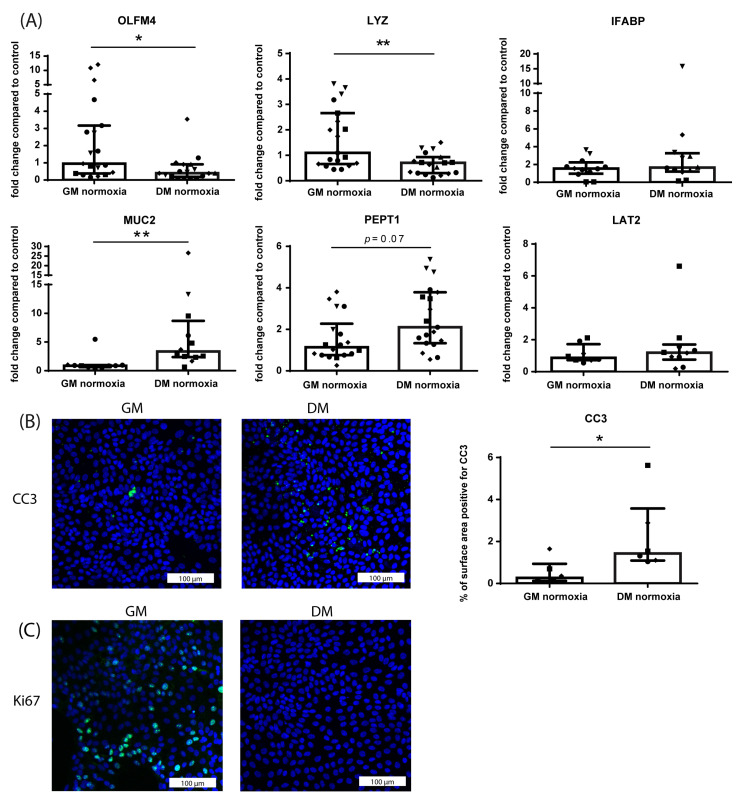
Effects of 60 h culturing with DM on HIO monolayers compared to GM-cultured controls. (**A**) mRNA expression of crypt cell markers OLFM4 (stem cells) and LYZ (Paneth cells), villus cell markers IFABP (enterocytes), MUC2 (Goblet cells) and PEPT1 (enterocytes) and LAT2 (amino acid transporter). Data are reported as relative expression compared to GM (set at 1) and displayed as median with interquartile range. Results were obtained from three different HIO donors (depicted by different data point symbols, one symbol type per donor). * *p* ≤ 0.05, ** *p* ≤ 0.01. (**B**) Representative images of immunofluorescence staining of CC3 in HIO monolayers cultured with GM or DM for 60 h and analysis of the % surface area positive for CC3 in both groups. Data are displayed as median with interquartile range. Results were obtained from three different HIO donors (depicted by different data point symbols, one symbol type per donor). Scale bars indicate 100 µm. * *p* ≤ 0.05. (**C**) Representative images of immunofluorescence staining of Ki67 in HIO monolayers cultured with GM or DM for 60 h. Results were obtained from three different HIO donors. Scale bars indicate 100 µm. Abbreviations: OLFM4—olfactomedin 4; LYZ—lysozyme; IFABP—intestinal fatty acid binding protein; MUC2—mucin 2; PEPT1—peptide transporter 1; LAT2—L-type amino acid transporter 2; CC3—cleaved caspase 3.

To induce differentiation towards a villus-like phenotype, HIO monolayers were cultured with DM for 60 h. This decreased the mRNA expression of crypt cell markers OLFM4 (stem cell maker; *p* ≤ 0.05) and LYZ (Paneth cell marker, *p* ≤ 0.01) compared to GM-cultured controls (Figure 2A). Concomitantly, mRNA expression of villus cell markers MUC2 (goblet cell marker; *p* ≤ 0.01) was increased, and PEPT1 tended to be increased (di-/tri-peptide transporter, enterocyte marker; *p* = 0.07) in DM-cultured monolayers compared to GM-cultured monolayers (Figure 2A). No differences were observed in mRNA expression of IFABP (enterocyte marker) and LAT2 (neutral amino acid transporter) in DM- versus GM-cultured monolayers (Figure 2A). DM culturing increased intestinal epithelial apoptosis (CC3 immunoreactivity) compared to GM-cultured controls (*p* ≤ 0.05; Figure 2B). In addition, after 60 h of DM culturing, intestinal epithelial proliferation (Ki67 immunoreactivity) was completely lost, whereas proliferating cells were still present in GM-cultured HIO monolayers (Figure 2C).

#### 3.1.2. Effect of Hypoxia on Crypt-like HIO Monolayers and 3D HIO

**Figure 3:**
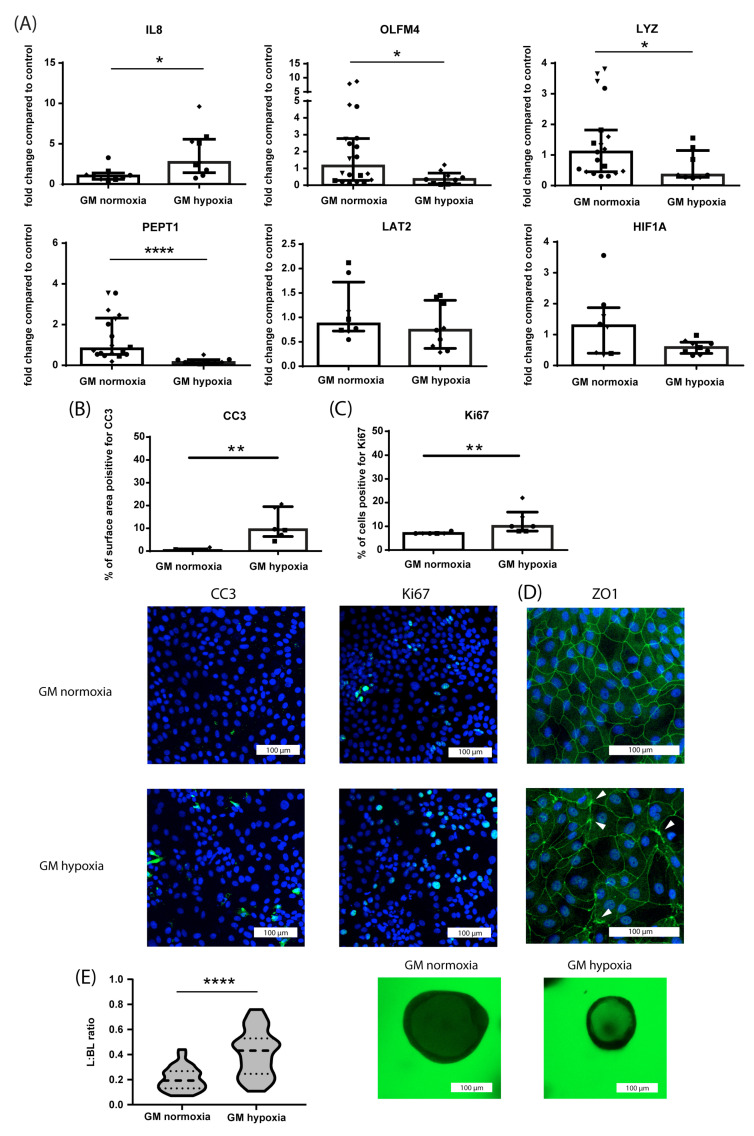
Effects of hypoxia on crypt-like (GM-cultured) HIO (monolayer- and 3D-cultured). (**A**) mRNA expression of IL8 (pro-inflammatory cytokine), OLFM4 (stem cells), LYZ (Paneth cells), PEPT1 (di/tri-peptide transporter), LAT2 (amino acid transporter) and HIF1A (hypoxia-regulated transcription factor). Data are reported as relative expression compared to normoxia (set at 1) and displayed as median with interquartile range. Results were obtained from three different HIO donors (depicted by different data point symbols, one symbol type per donor). * *p* ≤ 0.05, **** *p* ≤ 0.0001. (**B**) Analysis of the % of surface area positive for CC3 and representative images from immunofluorescence staining of CC3 in crypt-like HIO monolayers exposed to hypoxia for 24 h compared to normoxic controls. Data are displayed as median with interquartile range. Results were obtained from three different HIO donors (depicted by different data point symbols, one symbol type per donor). Scale bars indicate 100 µm. ** *p* ≤ 0.01. (**C**) Analysis of the % of cells positive for Ki67 and representative images from immunofluorescence staining of Ki67 in crypt-like HIO monolayers exposed to hypoxia for 24 h compared to normoxic controls. Data are displayed as median with interquartile range. Results were obtained from three different HIO donors (depicted by different data point symbols, one symbol type per donor). Scale bars indicate 100 µm. ** *p* ≤ 0.01. (**D**) Representative images from immunofluorescence staining of ZO1 in crypt-like HIO monolayers exposed to hypoxia for 24 h compared to normoxic controls. Results were obtained from three different HIO donors. Scale bars indicate 100 µm. (**E**) FITC-D4 paracellular barrier assay with representative images of crypt-like 3D HIO exposed to hypoxia for 16 h compared to controls. FITC-D4 fluorescence intensity is expressed as a luminal (L) to basolateral (BL) ratio. Per group, at least 10 HIO were measured in three different donors. Scale bars indicate 100 µm. **** *p* ≤ 0.0001. Abbreviations: IL8—interleukin 8; OLFM4—olfactomedin 4; LYZ—lysozyme; PEPT1—peptide transporter 1; LAT2—L-type amino acid transporter 2; HIF1A—hypoxia inducible factor 1 alpha; CC3—cleaved caspase 3; ZO1—zona occludens 1.

Hypoxia was used to induce a diseased phenotype in the crypt-like HIO since it is an important factor contributing to the pathogenesis of IBD [51], IRI [52] and NEC [53]. Exposure of crypt-like (GM-cultured) HIO monolayers to hypoxia (1% O_2_) for 24 h increased mRNA expression of the pro-inflammatory cytokine IL8 and decreased the mRNA expression of OLFM4 (*p* ≤ 0.05), LYZ (*p* ≤ 0.05) and PEPT1 (*p* ≤ 0.0001) compared to normoxic controls (Figure 3A). Furthermore, mRNA expression of LAT2 and Hypoxia Inducible Factor 1 alpha (HIF1A, hypoxia-regulated transcription factor) were unaltered (Figure 3A). Importantly, however, mRNA expression of HIF1A peaked at earlier time points following the onset of hypoxia (Supplementary Figure S3). Exposure to hypoxia increased intestinal epithelial apoptosis (CC3 immunoreactivity, Figure 3B) and proliferation (Ki67 immunoreactivity, Figure 3C) in crypt-like HIO monolayers compared to normoxic controls (*p* ≤ 0.01, Figure 3D), indicating increased epithelial cell turnover. In addition, 24 h exposure to hypoxia caused disruption of ZO1 protein expression (Figure 3D), characterized by unequal division of ZO1 protein across the cell–cell surface and focal ZO1 accumulation. Last, after 16 h of hypoxia, the paracellular barrier function of 3D crypt-like HIO was diminished compared to normoxic controls (increased L:BL ratio in FITC-D4 paracellular barrier assay, Figure 3E). For this experiment, 16 h of hypoxia was chosen as this gave a steady loss of barrier function in the hypoxic organoids, while normoxic controls retained a good barrier function, concurrently allowing for pre-incubation of the nutritional components before the start of hypoxia within a 24 h study period.

#### 3.1.3. Effect of Hypoxia on Villus-like HIO Monolayers

**Figure 4:**
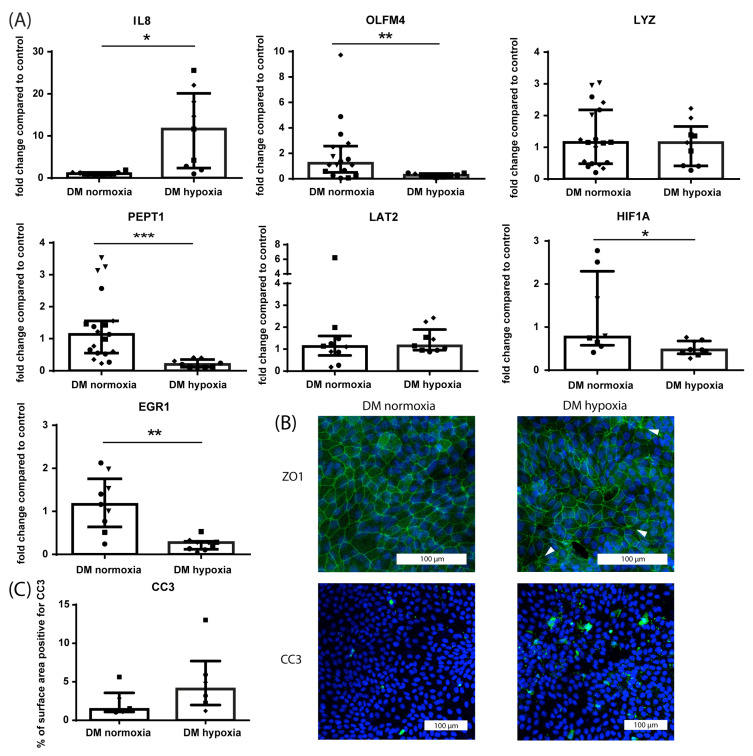
Effects of hypoxia on villus-like (DM-cultured) HIO (monolayer-cultured). (**A**) mRNA expression of IL8 (pro-inflammatory cytokine), OLFM4 (stem cells), LYZ (Paneth cells), PEPT1 (di/tri-peptide transporter), LAT2 (amino acid transporter) and HIF1A (hypoxia-regulated transcription factor) following 48 h of hypoxia (1% O_2_) in villus-like HIO monolayers. Data are reported as relative expression compared to normoxia (set at 1) and displayed as median with interquartile range. Results were obtained from three different HIO donors (depicted by different data point symbols, one symbol type per donor). * *p* ≤ 0.05, ** *p* ≤ 0.01, *** *p* ≤ 0.001. (**B**) Representative images from immunofluorescence staining of ZO1 in villus-like HIO monolayers exposed to hypoxia for 24 h compared to normoxic controls. Results were obtained from three different HIO donors. Scale bars indicate 100 µm. (**C**) Analysis of the % of surface area positive for CC3 and representative images from immunofluorescence staining of CC3 in villus-like HIO monolayers exposed to hypoxia for 24 h compared to normoxic controls. Data are displayed as median with interquartile range. Results were obtained from three different HIO donors (depicted by different data point symbols, one symbol type per donor). Scale bars indicate 100 µm. Abbreviations: IL8—interleukin 8; OLFM4—olfactomedin 4; LYZ—lysozyme; PEPT1—peptide transporter 1; LAT2—L-type amino acid transporter 2; HIF1A—hypoxia inducible factor 1 alpha; ZO1—zona occludens 1; CC3—cleaved caspase 3.

Hypoxia was used to induce a diseased phenotype in villus-like HIO as well. A longer hypoxia period of 48 h was chosen for DM-cultured HIO monolayers compared to GM-cultured HIO monolayers, as DM-cultured HIO were observed to be less sensitive to the effects of hypoxia (unpublished observations). In villus-like HIO (DM-cultured), exposure to hypoxia (1% O_2_) for 48 h increased the mRNA expression of IL8 (*p* ≤ 0.05) and decreased the mRNA expression of OLFM4 (*p* ≤ 0.01) and PEPT1 (*p* ≤ 0.001) compared to normoxic controls (Figure 4A). Additionally, mRNA expression of HIF1A (*p* ≤ 0.05) was decreased after 48 h of hypoxia compared to normoxic controls (Figure 4A), which may result from the effect of negative feedback loops on its expression following prolonged hypoxia [54,55]. Furthermore, mRNA expression of LYZ and LAT2 was unaltered following hypoxia at the studied time point (Figure 4A). Exposing villus-like HIO monolayers to hypoxia for 24 h mildly disrupted ZO1 protein expression (Figure 4B), characterized by focal ZO1 protein accumulation. In addition, 24 h of hypoxia caused an increase in intestinal epithelial apoptosis (CC3 immunoreactivity), although this increase did not reach statistical significance (Figure 4C).

#### 3.1.4. Differential Effect of Hypoxia on Crypt-like and Villus-like HIO Monolayers and 3D HIO

**Figure 5:**
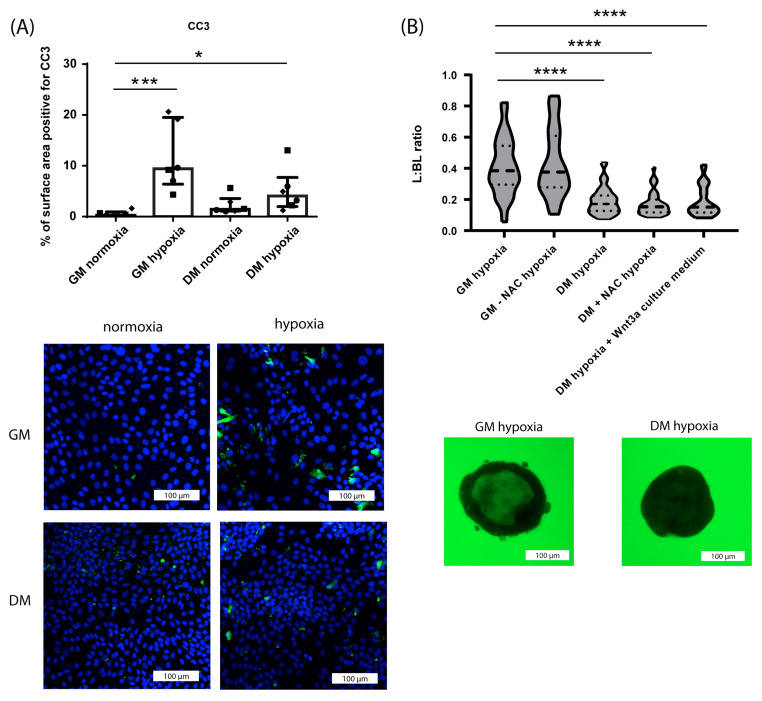
Differential effect of hypoxia on crypt-like and villus-like HIO (3D- and monolayer-cultured). (**A**) Analysis of the % of surface area positive for CC3 and representative images from immunofluorescence staining of CC3 in crypt-like and villus-like HIO monolayers exposed to hypoxia for 24 h compared to normoxic controls. Data are displayed as median with interquartile range. Results were obtained from three different HIO donors (depicted by different data point symbols, one symbol type per donor). Scale bars indicate 100 µm. (**B**) FITC-D4 paracellular barrier assay of crypt-like (GM-cultured) and villus-like (DM-cultured) 3D HIO exposed to hypoxia for 16 h, in presence or absence of NAC and following replacement of the surplus of AdDF+++ in DM by the DMEM medium used for the production of Wnt3a-conditioned medium. Representative images of crypt-like (GM-cultured) and villus-like (DM-cultured) 3D HIO exposed to hypoxia for 16 h. FITC-D4 fluorescence intensity is expressed as a luminal (L) to basolateral (BL) ratio. Per group, 15 HIO were measured in 3 different donors. Scale bars indicate 100 µm. * *p* ≤ 0.5, *** *p* ≤ 0.001, **** *p* ≤ 0.0001. Abbreviations: CC3—cleaved caspase 3.

Villus-like HIO, both 3D and monolayer-cultured, displayed less damage following exposure to hypoxia than crypt-like HIO. Both in crypt-like and villus-like HIO monolayers, hypoxia led to increased intestinal epithelial cell death (CC3 immunoreactivity) (Figure 5A). However, the increase was less pronounced in villus-like HIO monolayers than in crypt-like HIO monolayers (Figure 5A). Comparably, 16 h of hypoxia exposure caused substantially less loss of paracellular barrier function in villus-like 3D HIO than in crypt-like 3D HIO (Figure 5B). This difference in vulnerability to hypoxia was not abrogated by the removal of the anti-oxidant NAC from GM, the addition of NAC to DM, or the replacement of the surplus of AdDF+++ in DM by the DMEM medium used for the production of Wnt3a-conditioned medium (Figure 5B).

### 3.2. Comprehensive Screening of Whey Protein Fractions

#### 3.2.1. Effect of WPI, DH28 and DH51 on Crypt-like HIO (Monolayer- and 3D-Cultured) in a Healthy Setting (Normoxia)

**Figure 6:**
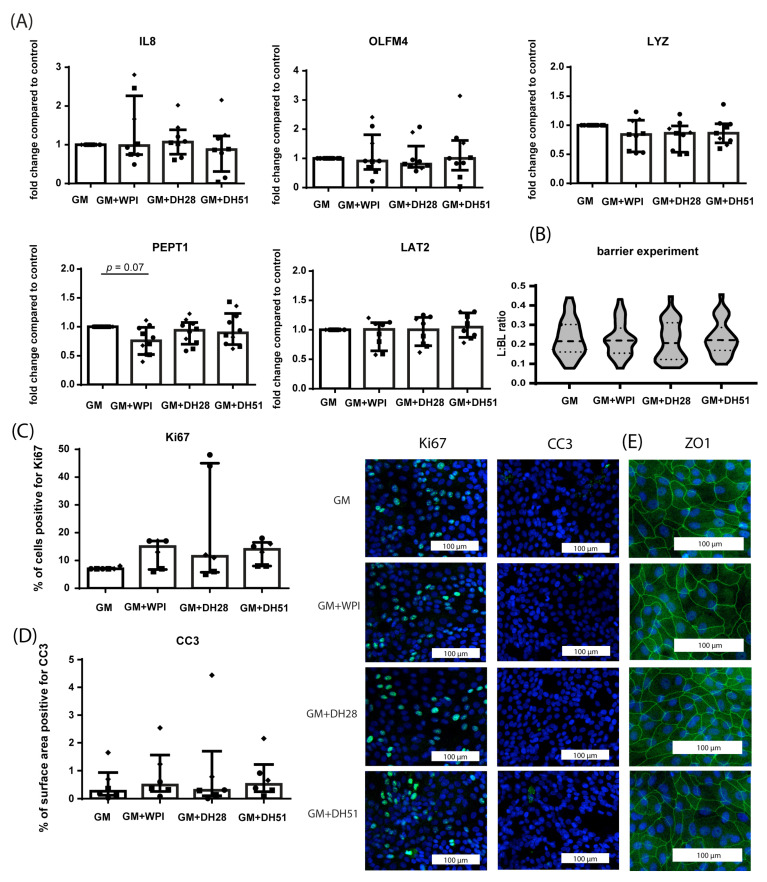
Effect of WPI, DH28, and DH51 on crypt-like HIO (monolayer- and 3D-cultured) in a healthy setting (normoxia). (**A**) mRNA expression of IL8 (pro-inflammatory cytokine), OLFM4 (stem cells), LYZ (Paneth cells), PEPT1 (di/tri-peptide transporter) and LAT2 (amino acid transporter) in crypt-like HIO monolayers following 24 h exposure to WPI, DH28, or DH51 compared to control. Data are reported as relative expression compared to control (set at 1) and displayed as median with interquartile range. Results were obtained from three different HIO donors (depicted by different data point symbols, one symbol type per donor). (**B**) FITC-D4 paracellular barrier assay of crypt-like 3D HIO exposed to WPI, DH28, or DH51 for 24 h compared to controls. FITC-D4 fluorescence intensity is expressed as a luminal (L) to basolateral (BL) ratio. Per group, 10 HIO were measured in three different donors. (**C**) Analysis of the % of cells positive for Ki67 and representative images from immunofluorescence staining of Ki67 in crypt-like HIO monolayers exposed to WPI, DH28, or DH51 for 24 h compared to controls. Data are displayed as median with interquartile range. Results were obtained from three different HIO donors (depicted by different data point symbols, one symbol type per donor). Scale bars indicate 100 µm. (**D**) Analysis of the % of surface area positive for CC3 and representative images from immunofluorescence staining of CC3 in crypt-like HIO monolayers exposed to WPI, DH28, or DH51 for 24 h compared to controls. Data are displayed as median with interquartile range. Results were obtained from three different HIO donors (depicted by different data point symbols, one symbol type per donor). Scale bars indicate 100 µm. (**E**) Representative images from immunofluorescence staining of ZO1 in crypt-like HIO monolayers exposed to WPI, DH28, or DH51 for 24 h compared to controls. Results were obtained from three different HIO donors. Scale bars indicate 100 µm. Abbreviations: IL8—interleukin 8; OLFM4—olfactomedin 4; LYZ—lysozyme; PEPT1—peptide transporter 1; LAT2—L-type amino acid transporter 2; CC3—cleaved caspase 3, ZO1—zona occludens 1; WPI—whey protein isolate; DH28—whey protein hydrolysate with 27.7% degree of hydrolysis; DH51—whey protein hydrolysate with 50.9% degree of hydrolysis.

In a healthy setting (normoxia), 24 h exposure to the different whey fractions of crypt-like HIO monolayers did not alter the mRNA expression of IL8, OLFM4, LYZ, or LAT2 (Figure 6A). PEPT1 mRNA expression tended to be decreased by exposure to WPI, but this difference did not reach statistical significance (*p* = 0.07) (Figure 6A). Paracellular barrier function of 3D HIO was unaltered by the different whey fractions under normoxic conditions (Figure 6B). In addition, exposure to the different whey fractions did not alter the amount of proliferating intestinal epithelial cells (Ki67 immunoreactivity) (Figure 6C), the level of intestinal epithelial apoptosis (Figure 6D), or ZO1 protein expression (Figure 6E).

#### 3.2.2. Effect of WPI, DH28 and DH51 on Villus-like HIO (Monolayer- and 3D-Cultured) in a Healthy Setting (Normoxia)

**Figure 7:**
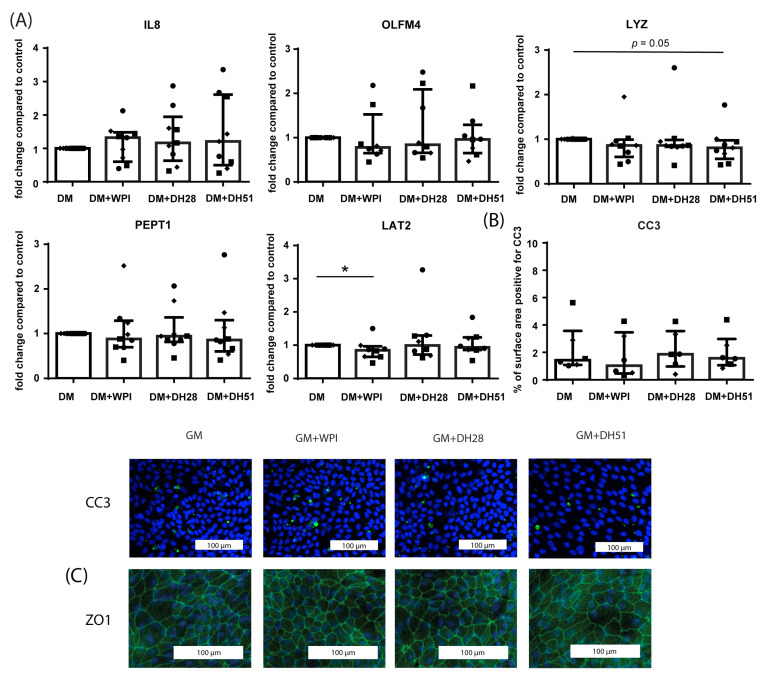
Effect of WPI, DH28 and DH51 on villus-like HIO (monolayer-cultured) in a healthy setting (normoxia). (**A**) mRNA expression of IL8 (pro-inflammatory cytokine), OLFM4 (stem cells), LYZ (Paneth cells), PEPT1 (di/tri-peptide transporter) and LAT2 (amino acid transporter) in villus-like HIO monolayers following 24 h exposure to WPI, DH28, or DH51 compared to control. Data are reported as relative expression compared to control (set at 1) and displayed as median with interquartile range. Results were obtained from 3 different HIO donors (depicted by different data point symbols, one symbol type per donor). * *p* ≤ 0.05 (**B**) Analysis of the % of surface area positive for CC3 and representative images from immunofluorescence staining of CC3 in villus-like HIO monolayers exposed to WPI, DH28 or DH51 for 24 h compared to controls. Data are displayed as median with interquartile range. Results were obtained from 3 different HIO donors (depicted by different data point symbols, one symbol type per donor). Scale bars indicate 100 µm. (**C**) Representative images from immunofluorescence staining of ZO1 in villus-like HIO monolayers exposed to WPI, DH28, or DH51 for 24 h compared to controls. Results were obtained from 3 different HIO donors. Scale bars indicate 100 µm. Abbreviations: IL8—interleukin 8; OLFM4—olfactomedin 4; LYZ—lysozyme; PEPT1—peptide transporter 1; LAT2—L-type amino acid transporter 2; CC3—cleaved caspase 3; WPI—whey protein isolate; DH28—whey protein hydrolysate with 27.7% degree of hydrolysis; DH51—whey protein hydrolysate with 50.9% degree of hydrolysis.

Exposing villus-like HIO monolayers for 24 h to WPI, DH28, or DH51 did not alter the mRNA expression of IL8, OLFM4, or PEPT1 (Figure 7A) compared to controls in a healthy setting (normoxia). LAT2 mRNA expression was moderately but statistically significantly decreased by WPI (*p* ≤ 0.05, Figure 7A). In addition, LYZ mRNA expression tended to be decreased by DH51 (*p* = 0.05, Figure 7A). Exposure to the different whey fractions in a healthy setting (normoxia) did not alter the level of intestinal epithelial apoptosis (Figure 7B) or ZO1 protein expression (Figure 7C).

#### 3.2.3. Effect of WPI, DH28 and DH51 on Crypt-like HIO (Monolayer- and 3D-Cultured) in a Diseased Setting (Hypoxia)

**Figure 8:**
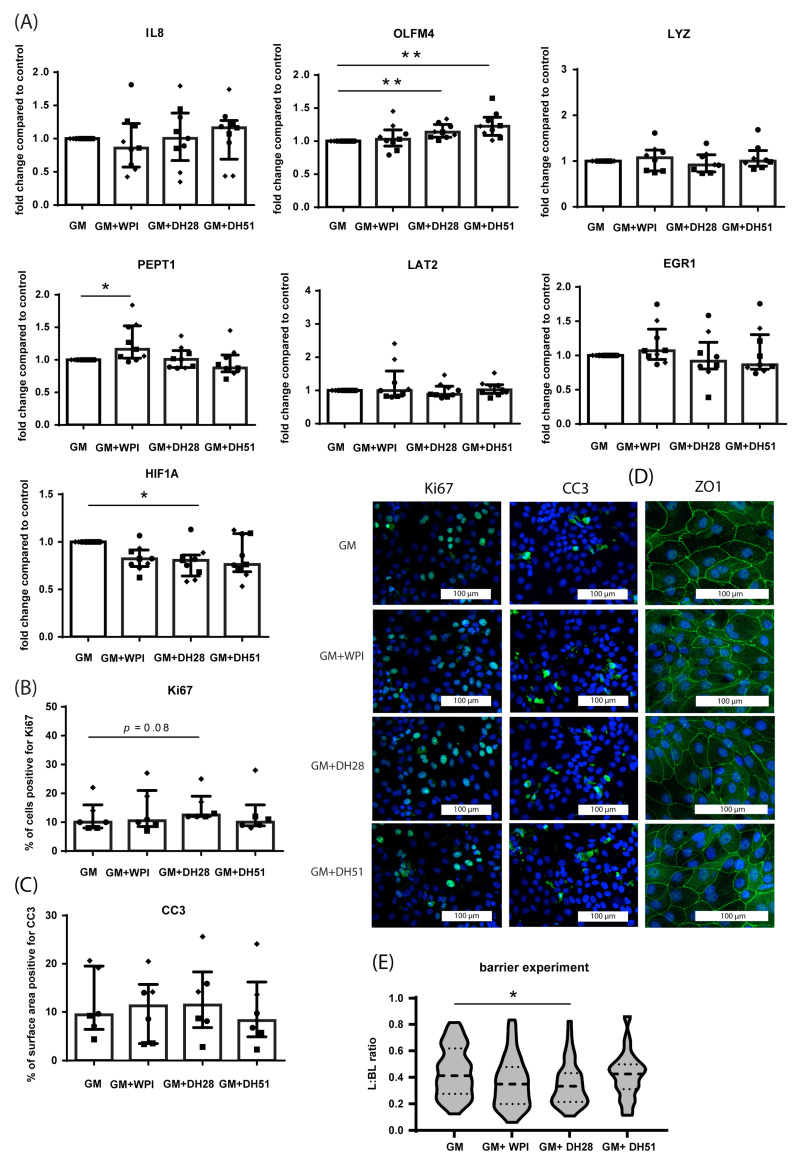
Effect of WPI, DH28, and DH51 on crypt-like HIO (monolayer- and 3D-cultured) in a diseased setting (hypoxia). (**A**) mRNA expression of IL8 (pro-inflammatory cytokine), OLFM4 (stem cells), LYZ (Paneth cells), PEPT1 (di/tri-peptide transporter), LAT2 (amino acid transporter) and HIF1A (hypoxia-regulated transcription factor) in crypt-like HIO monolayers exposed to hypoxia (24 h) and WPI, DH28, or DH51 (24 h) compared to control. Data are reported as relative expression compared to control (set at 1) and displayed as median with interquartile range. Results were obtained from 3 different HIO donors (depicted by different data point symbols, one symbol type per donor). * *p* ≤ 0.05, ** *p* ≤ 0.01. (**B**) Analysis of the % of cells positive for Ki67 and representative images from immunofluorescence staining of Ki67 in crypt-like HIO monolayers exposed to hypoxia (24 h) and WPI, DH28, or DH51 (24 h) compared to controls. Data are displayed as median with interquartile range. Results were obtained from three different HIO donors (depicted by different data point symbols, one symbol type per donor). Scale bars indicate 100 µm. (**C**) Analysis of the % of surface area positive for CC3 and representative images from immunofluorescence staining of CC3 in crypt-like HIO monolayers exposed to hypoxia (24 h) and WPI, DH28, or DH51 (24 h) compared to controls. Data are displayed as median with interquartile range. Results were obtained from three different HIO donors (depicted by different data point symbols, one symbol type per donor). Scale bars indicate 100 µm. (**D**) Representative images from immunofluorescence staining of ZO1 in crypt-like HIO monolayers exposed to hypoxia (24 h) and WPI, DH28, or DH51 (24 h) compared to controls. Results were obtained from three different HIO donors. Scale bars indicate 100 µm. (**E**) FITC-D4 paracellular barrier assay of crypt-like 3D HIO exposed to hypoxia (16 h) and WPI, DH28, or DH51 (24 h) compared to controls. FITC-D4 fluorescence intensity is expressed as a luminal (L) to basolateral (BL) ratio. Per group, 15 HIO were measured in three different donors. * *p* ≤ 0.05 Abbreviations: IL8—interleukin 8; OLFM4—olfactomedin 4; LYZ—lysozyme; PEPT1—peptide transporter 1; LAT2—L-type amino acid transporter 2; HIF1A—hypoxia inducible factor 1 alpha; ZO1—zona occludens 1; CC3—cleaved caspase 3; WPI—whey protein isolate; DH28—whey protein hydrolysate with 27.7% degree of hydrolysis; DH51—whey protein hydrolysate with 50.9% degree of hydrolysis.

In a diseased setting (24 h exposure to hypoxia), 24 h exposure to DH28 and DH51 increased the mRNA expression of stem cell marker OLFM4 (both *p* ≤ 0.01) (Figure 8A) in crypt-like HIO monolayers. Exposure to DH28 concomitantly decreased the mRNA expression of HIF1A (*p* ≤ 0.05) (Figure 8A). In addition, exposure to WPI increased the mRNA expression of PEPT1 (*p* ≤ 0.05) (Figure 8A). Furthermore, mRNA expression of IL8, LYZ and LAT2 was unaffected by exposure to WPI, DH28, or DH51 in crypt-like hypoxic HIO monolayers. The amount of proliferating intestinal epithelial cells (Ki67 immunoreactivity) tended to be increased by exposure to DH28, although this increase did not reach statistical significance (*p* = 0.08) (Figure 8B). The level of intestinal epithelial apoptosis (CC3 immunoreactivity) was not changed by the addition of WPI, DH28, or DH51 (Figure 8C) to the GM, nor was the ZO1 protein expression (Figure 8D). Importantly, however, paracellular barrier function was improved by exposure to DH28 (*p* ≤ 0.05) (Figure 8E).

#### 3.2.4. Effect of WPI, DH28 and DH51 on Villus-like HIO (Monolayer- and 3D-Cultured) in a Diseased Setting (Hypoxia)

**Figure 9:**
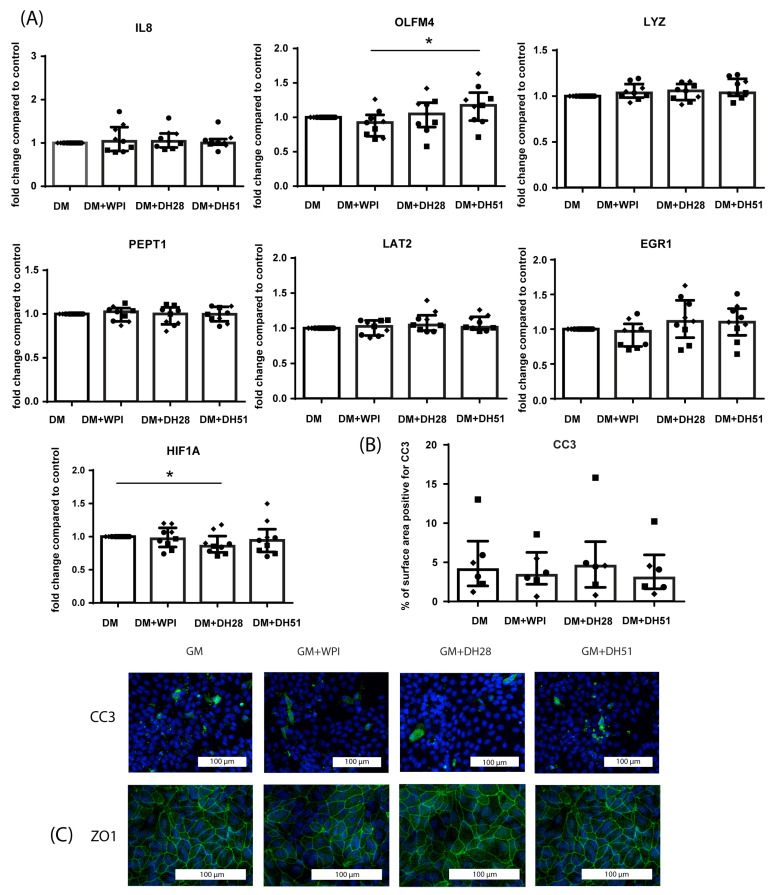
Effect of WPI, DH28 and DH51 on villus-like HIO (monolayer-cultured) in a diseased setting (hypoxia). (**A**) mRNA expression of IL8 (pro-inflammatory cytokine), OLFM4 (stem cells), LYZ (Paneth cells), PEPT1 (di/tri-peptide transporter), LAT2 (amino acid transporter) and HIF1A (hypoxia-regulated transcription factor) in villus-like HIO monolayers exposed to hypoxia (48 h) and WPI, DH28 or DH51 (48 h) compared to control. Data are reported as relative expression compared to control (set at 1) and displayed as median with interquartile range. Results were obtained from 3 different HIO donors (depicted by different data point symbols, one symbol type per donor). * *p* ≤ 0.05. (**B**) Analysis of the % of surface area positive for CC3 and representative images from immunofluorescence staining of CC3 in villus-like HIO monolayers exposed to hypoxia (24 h) and WPI, DH28, or DH51 (24 h) compared to controls. Data are displayed as median with interquartile range. Results were obtained from 3 different HIO donors (depicted by different data point symbols, one symbol type per donor). Scale bars indicate 100 µm. (**C**) Representative images from immunofluorescence staining of ZO1 in villus-like HIO monolayers exposed to hypoxia (24 h) and WPI, DH28, or DH51 (24 h) compared to controls. Results were obtained from 3 different HIO donors. Scale bars indicate 100 µm. Abbreviations: IL8—interleukin 8; OLFM4—olfactomedin 4; LYZ—lysozyme; PEPT1—peptide transporter 1; LAT2—L-type amino acid transporter 2; HIF1A—hypoxia inducible factor 1 alpha; CC3—cleaved caspase 3; ZO1—zona occludens 1; WPI—whey protein isolate; DH28—whey protein hydrolysate with 27.7% degree of hydrolysis; DH51—whey protein hydrolysate with 50.9% degree of hydrolysis.

In a diseased setting (48 h exposure to hypoxia), 48 h exposure to DH51 increased the mRNA expression of stem cell marker OLFM4 (*p* ≤ 0.05) (Figure 9A) in villus-like HIO monolayers. HIF1A mRNA expression was reduced by exposure to DH28 (*p* ≤ 0.05) (Figure 9A). The different whey fractions did not alter the mRNA expression of IL8, LYZ, PEPT1 and LAT2 (Figure 9A). The level of intestinal epithelial apoptosis (CC3 immunoreactivity) was not changed by the addition of WPI, DH28, or DH51 (Figure 9B) to the DM for 24 h in a diseased setting (24 h hypoxia). Last, ZO1 protein expression was not affected by the administration of WPI, DH28, or DH51 (24 h) during hypoxia (24 h) (Figure 9C).

#### 3.2.5. Effect of WPI, DH28 and DH51 on T Cell Subsets and T Cell Proliferation

**Figure 10:**
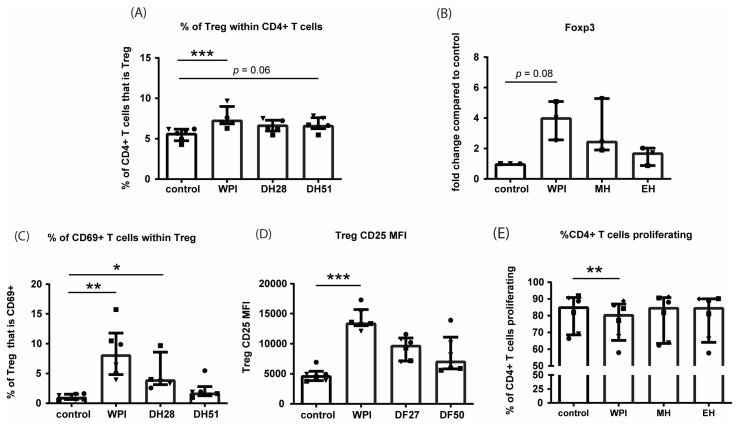
Number and CD25 and CD69 expression of CD4+CD25^high^CD127^low^ Treg in unactivated PBMCs, and proliferation of CD4+ T cells in T cell-activated PBMCs following incubation with WPI, DH28 or DH51. (**A**) The percentage of Treg (CD4+CD25^high^CD127^low^ T cells) within the population of CD4+ T cells in unactivated PBMCs incubated with or without WPI, DH28, or DH51 for 5 days. Data are displayed as median with interquartile range. *** *p* ≤ 0.001 Results were obtained from three different PBMC donors (depicted by different data point symbols, one symbol type per donor). (**B**) mRNA expression of Foxp3 in unactivated PBMCs incubated with or without WPI, DH28, or DH51 for 5 days. Data are reported as relative expression compared to control (set at 1) and displayed as median with interquartile range. Results were obtained from four different PBMC donors (depicted by different data point symbols, one symbol type per donor). ** *p* ≤ 0.01 (**C**) Percentage of CD69+ T cells within the population of Treg in unactivated PBMCs incubated with or without WPI, DH28, or DH51 for 5 days. Data are displayed as median with interquartile range. * *p* ≤ 0.05, ** *p* ≤ 0.01 Results were obtained from three different PBMC donors (depicted by different data point symbols, one symbol type per donor). (**D**) CD25 expression (MFI) of Treg in unactivated PBMCs incubated with or without WPI, DH28, or DH51 for 5 days. Data are displayed as median with interquartile range. *** *p* ≤ 0.001 Results were obtained from three different PBMC donors (depicted by different data point symbols, one symbol type per donor). (**E**) The percentage of CD4+ cells proliferating in T cell-activated PBMCs incubated with or without WPI, DH28, or DH51 for 5 days. Results were obtained from four different PBMC donors (depicted by different data point symbols, one symbol type per donor). Data are displayed as median with interquartile range. ** *p* ≤ 0.01 Abbreviations: CD4—cluster of differentiation 4; Foxp3—forkhead box P3; CD25—cluster of differentiation 25; CD69—cluster of differentiation 69; CD127—cluster of differentiation 127; WPI—whey protein isolate; DH28—whey protein hydrolysate with 27.7% degree of hydrolysis; DH51—whey protein hydrolysate with 50.9% degree of hydrolysis; MFI—mean fluorescent intensity.

We studied the effect of WPI, DH28, and DH51 on regulatory T cells (Treg) with flow cytometric analysis of unactivated PBMCs cultured with or without the different experimental fractions for five days (Supplementary Figure S4). WPI increased the percentage of Treg (CD4+CD25^high^CD127^low^ T cells) within the population of CD4+ T cells (*p* ≤ 0.001, Figure 10A). This finding was supported by an increased FoxP3 mRNA expression in this group (*p* ≤ 0.01, Figure 10B). In addition, incubation with WPI increased the percentage of CD69+ T cells within the population of Treg and increased the CD25 expression (MFI) of Treg (*p* ≤ 0.01 and *p* ≤ 0.001, respectively, Figure 10C,D). The percentage of CD69+ T cells in Treg was also increased by incubation with DH28 (*p* ≤ 0.05, Figure 10C). These findings prompted us to study the effects of WPI, DH28, and DH51 on T cell proliferation of T cell- activated PBMCs (Supplementary Figure S5). Incubation with WPI, but not DH28 or DH51, decreased the percentage of CD4+ T cells proliferating (*p* ≤ 0.01) (Figure 10E, Supplementary Figure S5). CD8+ T cell proliferation also tended to be decreased by exposure to WPI (*p* = 0.08, Supplementary Figure S5).

#### 3.2.6. Effect of WPI, DH28 and DH51 on PBMC Cytokine Expression and Activation Makers

**Figure 11:**
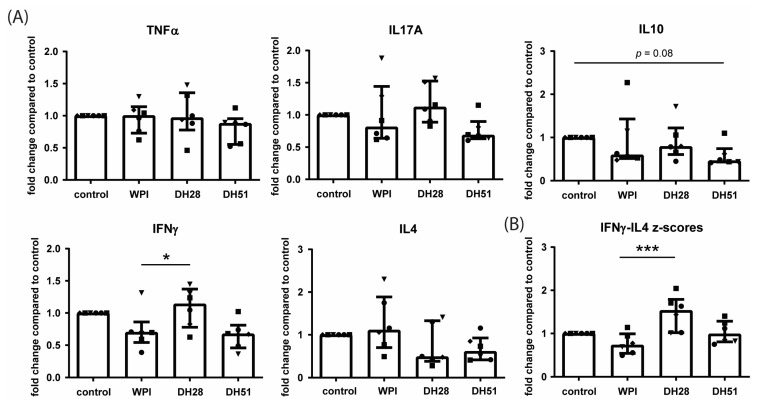
mRNA expression of various cytokines in T cell-activated PBMCs following incubation with WPI, DH28, or DH51. (**A**) mRNA expression of TNFα (Th1 cytokine), IL17A (Th17 cytokine), IL-10 (regulatory T cell cytokine), IFNγ (Th1 cytokine) and IL4 (Th2 cytokine) in T cell-activated PBMCs incubated with or without WPI, DH28 or DH51 for 5 days. Data are reported as relative expression compared to control (set at 1) and displayed as median with interquartile range. Results were obtained from four different PBMC donors (depicted by different data point symbols, one symbol type per donor). * *p* ≤ 0.05. (**B**) mRNA expression of IFNγ relative to IL4 (IFNγ-IL4 z-scores, Th1-Th2 balance) in T cell- activated PBMCs incubated with or without WPI, DH28, or DH51 for 5 days. Data are reported as relative expression compared to control (set at 1) and displayed as median with interquartile range. Results were obtained from four different PBMC donors (depicted by different data point symbols, one symbol type per donor). *** *p* ≤ 0.001 Abbreviations: TNFα—tumor necrosis factor alpha; IL17A—interleukin 17A; IL10—interleukin 10; IFNγ—interferon gamma; IL4—interleukin 4; WPI—whey protein isolate; DH28—whey protein hydrolysate with 27.7% degree of hydrolysis; DH51—whey protein hydrolysate with 50.9% degree of hydrolysis.

Cytokine mRNA expression was studied in human PBMCs in which T cells were activated and were cultured with or without the different experimental fractions for 5 days. Upon T cell activation, incubation of PBMCs with DH51 tended to decrease the IL10 mRNA expression (Treg cytokine, *p* = 0.08) (Figure 11A). Incubation with DH28 increased the mRNA expression of IFNγ (Th1 cytokine, *p* ≤ 0.05, Figure 11A) and IFNγ relative to IL4 (IFNγ-IL4 z-score difference, Th1-Th2 balance, *p* ≤ 0.0001, Figure 11B) compared to incubation with WPI (Figure 11B). mRNA expression of TNFα (Th1 cytokine), IL17A (Th17 cytokine) and IL4 (Th2 cytokine) was not affected by incubation with WPI, DH28, or DH51 (Figure 11A).

#### 3.2.7. Effect of WPI, DH28 and DH51 on Four Microbial Strains

**Figure 12:**
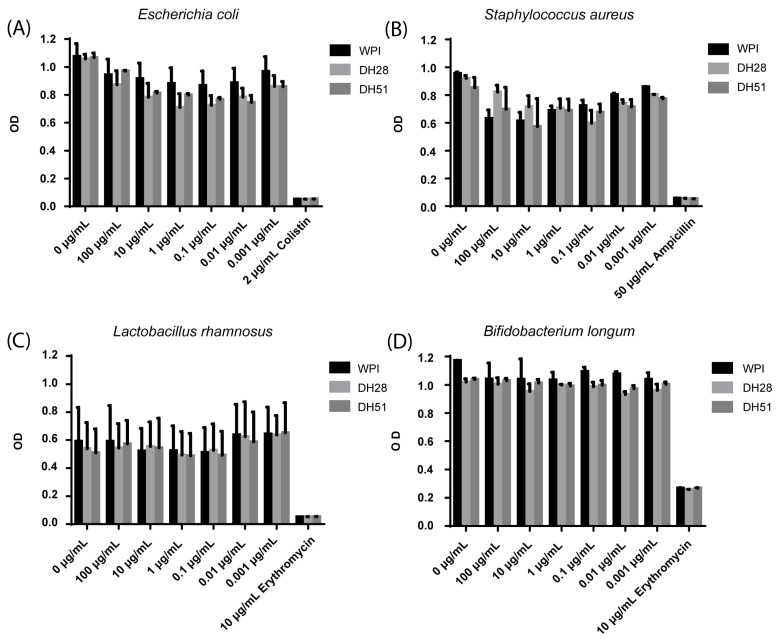
Absence of inhibition of growth of (**A**) *Escherichia coli*, (**B**) *Straphylococcus aureus*, (**C**) *Lactobacillus rhamnosus* and (**D**) *Bifodobacterium longum* by incubation with WPI, DH28, or DH51. Data are presented as mean and standard deviation of two independent measurements. Abbreviations: OD—optical density; WPI—whey protein isolate; DH28—whey protein hydrolysate with 27.7% degree of hydrolysis; DH51—whey protein hydrolysate with 50.9% degree of hydrolysis.

Effects of WPI, DH28 and DH51 of two pathogenic (*Escherichia coli* and *Staphylococcus aureus)* and two probiotics (*Lactobacillus rhamnosus* and *Bifidobacterium longum)* microbial strains were investigated with a serial dilutions growth inhibition test. WPI, DH28 and DH51 did not change the growth of *Escherichia coli, Staphylococcus aureus, Lactobacillus rhamnosus* and *Bifidobacterium longum* (Figure 12).

## 4. Discussion

In the current study, we extended a validated HIO monolayer culture model [41,42,56] to facilitate screening of the effects of (nutritional) substances, including whey protein fractions with different degrees of hydrolysis on undifferentiated and differentiated epithelial cells in healthy and a diseased state. In line with findings in earlier studies [41,42], replacing the normal growth medium (GM) with a differentiation medium (DM) changed the HIO phenotype from crypt-like to villus-like and, in accordance with the in vivo situation, completely blocked intestinal epithelial proliferation, thereby enabling nutritional intervention studies on the complete crypt-villus axis. Moreover, the HIO monolayer model allowed apical administration of the whey protein fractions. Exposure to hypoxia-induced a ‘diseased state’ in the HIO, characterized by intestinal epithelial inflammation (IL8 increase), increased intestinal epithelial apoptosis (CC3) and intestinal barrier loss, reflected by disturbed expression of the tight junction protein ZO1 and, in crypt-like 3D HIOs, a loss of paracellular barrier function. In addition, mRNA expression of the enterocyte marker PEPT1 (crypt-like and villus-like), the stem cell marker OLFM4 (crypt-like and villus-like) and Paneth cell marker LYZ (crypt-like) were reduced by exposure to hypoxia, which is considered to reflect cellular damage along the crypt-villus axis. Importantly, these combined characteristics are an essential part of the pathophysiology of several diseases affecting the intestine, such as IBD [51,57], IRI [52] and NEC [53,58], indicating the “diseased state” induced by hypoxia in this HIO model is potentially very relevant for studying the effect of nutritional interventions in a broad set of gastrointestinal diseases.

Interestingly, we observed that villus-like (DM-cultured) HIOs were less sensitive to the effects of hypoxia than crypt-like (GM-cultured) HIOs. This effect was already observed within 24 h after replacing GM with DM and was independent of the anti-oxidant NAC or the percentage of AdDF+++ in the medium. This suggests that the sensitivity of the HIO intestinal epithelial cells to hypoxia is, amongst others, determined by their differentiation status or, alternatively, by the presence of medium components directly related to differentiation status, such as Wnt3a. Differences in the energy metabolism of undifferentiated (i.e., stem cells and transit amplifying cells) versus differentiated intestinal epithelial cells are likely involved. Analogous to the Warburg effect in cancer cells, highly proliferating cells such as intestinal stem cells largely depend on aerobic glycolysis, whereas differentiated cells rely more on oxidative phosphorylation [59,60]. Wnt signaling appears to be an important factor in modifying this metabolic phenotype [60,61]. Importantly, differentiated organoids are described to have reduced oxygen consumption rates compared to undifferentiated organoids [62], which is not surprising considering the oxygen gradient along the crypt-villus axis and the physiological hypoxia that is present in the lumen of the intestine [63]. In addition, differences in the metabolic adaptation to hypoxia could be involved since Kip et al. previously showed on the protein level that crypt-like and villus-like HIOs differentially adapt their mitochondrial metabolism during hypoxia [64]. Recapitulating, it is important to take the differentiation status of HIOs into account when developing a hypoxia-based HIO model and to study nutritional interventions both in crypt-like and villus-like HIOs.

Following successful model development, this HIO model was used as a screening tool to study the effect of whey protein isolate (WPI) and two whey protein hydrolysates with a 28% degree of hydrolysis (DH28) and a 51% degree of hydrolysis (DH51) on the intestinal epithelium in health and disease.

A key finding is that the addition of DH28 protected the paracellular barrier function of crypt-like HIO during hypoxia. A possible explanation for this observation is DH28-driven increased intestinal epithelial proliferation. However, this should be interpreted with caution; although the increased proliferation following DH28 exposure may be biologically relevant, differences between the groups did not meet conventional levels of statistical significance (*p* = 0.08). Findings at the studied time point did not provide an alternative explanation for the protection of paracellular barrier function, however; we cannot rule out the possibility that factors such as reduction of apoptosis or improvement of tight junction integrity are involved at an earlier moment.

Improvement of paracellular barrier function could be causally linked to the reduced HIF1α mRNA expression that was detected in both crypt-like and villus-like HIOs following DH28 exposure. The HIF complex, including its hypoxia-inducible subunit HIF1α, is important for cellular adaptation to hypoxia via regulation of a broad range of cellular processes such as angiogenesis, an adaptation of metabolism and cell survival [65]. Besides, HIF1α signaling is important for the maintenance of intestinal barrier function via the regulation of tight junction integrity [66]. However, it can contribute to several disease mechanisms [67], such as intestinal inflammation and gut barrier loss [68], during prolonged and/or severe hypoxia. HIF1α signaling is directly controlled by cellular oxygen tension through the hydroxylation of HIF1α by oxygen-dependent prolyl-hydroxylase domain-containing enzymes (PHD) and subsequent protein breakdown [69]. Besides this direct regulation, several metabolic intermediates, including reactive oxygen species (ROS), increase HIF1α signaling, both via direct stabilization of HIF1α through PHD inhibition and via multiple indirect mechanisms [69,70]. Given the effect of ROS formation on HIF1α signaling, anti-oxidant peptides that have been described to be present in whey protein fractions are likely involved in the downregulation of HIF1α mRNA following DH28 exposure [30]. Alternatively, Sirtuin1 (SIRT1), as an important regulator of HIF1α signaling [71] and ROS [72], might be involved in the observed HIF1α reduction and barrier protection following exposure to DH28. This scenario is consistent with earlier findings where SIRT1-mediated protection of the intestinal barrier following LPS-induced inflammation in vitro was shown with concomitant downregulation of HIF1α protein expression and activity [73] and with upregulation of SIRT1 by whey protein [74].

Another evident observation is the increase in mRNA level of the stem cell marker OLFM4 following exposure to DH28 and DH51 in crypt-like (DH28 and DH51) and villus-like (DH51) HIOs under hypoxic conditions. Since the expression of other cell-specific markers, such as the Paneth cell marker LYZ did not change following exposure to the whey protein fractions, it is likely that the increase in OLFM4 mRNA represents an increased expression per cell rather than an increase in stem cell numbers. As OLFM4 mRNA expression was downregulated during hypoxia in both crypt-like and villus-like HIOs and OLFM4 is important for mucosal defense and acts as an anti-inflammatory molecule in IBD, these findings are potentially relevant for human translation [75,76,77].

On the immune cell level, exposure to WPI increased the percentage of Treg in the CD4+ T cell population in unactivated PBMCs, whereas this was not observed following exposure to DH28 or DH51. In addition, the expression of CD25 and CD69 by Treg was increased by incubation with WPI. Interestingly, CD69 expression was previously shown to increase IL10 expression by Treg and their suppressive capacities in mice [78,79]. In addition, adoptive cell transfer of CD69+ Treg, but not CD69− Treg or CD69+ Treg from IL10^−/−^ mice, reduced DSS-induced colitis severity in mice [78], suggesting WPI exposure may increase immune tolerance via induction of Treg with enhanced suppressive capacities. These findings prompted us to study the effects of WPI on T cell proliferation in T cell-activated PBMCs. In line with our Treg observations, WPI decreased the proliferation of CD4+ T cells following T cell activation. These findings confirm and extend findings in earlier studies; enteral administration of a bovine whey protein extract stimulated the generation of Treg in vivo in a murine asthma model [80], and oral treatment with a formula enriched with WPI improved oral tolerance to ovalbumin in mice [81]. Importantly, in these studies, the effects of whey protein could be largely attributed to the presence of TGFβ within the whey protein fraction [80,81]. TGFβ consists of a group of 12.7–25 kDa proteins that are virtually absent following hydrolyzation [81] and important for immune tolerance and Treg induction and function [82,83]. Thus, TGFβ could be an important factor in the observed changes in Treg numbers and expression profile and the reduced CD4+ T cell proliferation following WPI exposure in the current study. Of note, orally administered TGFβ was observed to retain its biological effect in intestinal mucosa and was associated with increased serum TGFβ levels in mice [84], which underscores the potential relevance of our in vitro findings for the in vivo situation. Interestingly, also T cell cytokine mRNA expression profile was differentially altered by exposure to the different whey protein fractions; DH28 exposure increased the relative expression of IFNγ to IL4 compared to WPI, suggesting DH28 exposure may shift the Th1-Th2 balance more towards a Th1 phenotype. If preserved in vivo, these in vitro findings could be of clinical relevance. Reduction of CD4+ T cell proliferation and induction of Treg by WPI administration could be used in infants in the prevention of NEC, which is characterized by the decreased proportion of Treg [85], or in the context of atopic diseases [86]. For adults, these findings might be relevant for several immune-mediated diseases with increasing prevalence, such as IBD or autoimmune diseases [87,88]. Additionally, shifting the Th1-Th2 balance towards a Th1 phenotype could be beneficial depending on both infants and adults based on several determinants, such as disease state [89,90,91], age [92] and atopic predisposition [93], which will be addressed in adjacent studies.

In this study, whey protein fractions were not found to have direct anti-microbial effects. Thus far, we have only investigated the effects of total whey protein fractions, which are comprised of a large array of individual whey peptides and proteins. We cannot rule out that specific whey peptides and/or proteins in our fractions do possess anti-microbial properties but that their concentration in the total solution was insufficient to observe an effect.

A limitation of the current study set-up is that the different whey protein fractions were administered basolaterally during the paracellular barrier function experiments, which could limit the bioactive effect of the whey protein fractions in case these depend on cellular uptake of di- or tri-peptides or on receptors that are solely expressed at the apical surface of the enterocyte. Another limitation is that, although the effects of the whey protein fractions on peripheral immune cells and microbial strains were investigated, experiments with interaction between intestinal epithelial cells, immune cells and microbial strains were not performed. One might argue that direct administration of the whey protein fractions to PBMCs limits its relevance to enteral administration of these fractions in vivo, in which PBMCs are separated from the intestinal lumen by the gut epithelium. However, a recent in vitro study using intestinal tissue of a 7-week-old pig in a Using chamber model demonstrated that, although in small amounts, whey oligopeptides with a molecular weight of up to ~4000 daltons (median ~1500 dalton) could pass the intestinal epithelial barrier [94]. In addition, several mechanisms of antigen sampling exist in the small intestine, such as uptake by dendritic cells protruding in the intestinal lumen, suggesting the direct influence of luminal content on local and systemic immune homeostasis [95]. To gain further insight into the relevance of our findings in this proof-of-concept study for the infant context, a head-to-head comparison with organoids derived from fetal or neonatal tissue and PBMCs derived from cord blood would be of added value. Last, in the current study set-up, the effects of gastrointestinal digestion were not accounted for, which should be addressed in future studies.

## 5. Conclusions

In summary, in the current study, we successfully developed an HIO model that enables screening of apically administered (nutritional) substances on intestinal epithelial cells, representing the full-crypt villus axis, during health and hypoxia-mediated intestinal inflammation. Comprehensive screening of different whey protein fractions revealed that protection of paracellular barrier function, downregulation of HIF1α and upregulation of stem cell marker OLFM4 during hypoxia-mediated inflammation, an increase of Treg numbers, alteration of their CD25 and CD69 expression profile and reduction of CD4+ T cell proliferation were differentially mediated by the different fractions, indicating that (degree of) hydrolysis determines their biological effects. Therefore, this should be taken into account when choosing specific nutritional products in a clinical/preventative setting and future studies assessing the biological effects of whey proteins/peptides. If also effective in an in vivo setting, WPI could be used for the prevention and treatment of immune-mediated diseases that may benefit from an increase of Treg or inhibition of CD4+ T cell proliferation. Given the protection of paracellular barrier function by DH28 during hypoxia-mediated intestinal inflammation, DH28 forms a promising candidate for preventing or treating several intestinal diseases and promoting intestinal health.

## Figures and Tables

**Table 1:** Primer sequences.

Primer	Forward	Reverse
β-actin	5′-ATTGCCGACAGGATGCAGAAG-3′	5′-TTGCTGATCCACATCTGCTGG-3′
GAPDH	5′-GGAAGCTCACTGGCATGGC-3′	5′-CCTGCTTCACCACCTTCTTG-3′
YWHAZ	5′-TGAACTCCCCTGAGAAAGCC-3′	5′-TCCGATGTCCACAATGTCAAGT-3′
CD3e	5′-TGCTGCTGGTTTACTACTGGA-3′	5′-GGATGGGCTCATAGTCTGGG-3′
IL8	5′-GCCGGAATACCTGGACTATGC-3′	5′-TTCCTTGGGGTCCAGACAGA-3′
OLFM4	5′-TGGACAGAGTGGAACGCTTG-3′	5′-TCAGAGCCACGATTTCTCGG-3′
LYS	5′-GATAACATCGCTGATGCTGTAGCT-3′	5′-CATGCCACCCATGCTCTAATG-3′
IFABP	5′-ACGGACAGACAATGGAAACGA-3′	5′-ACTGTGCGCCAAGAATAATGC-3′
MUC2	5′-CTACTGGTGTGAGTCCAAGG-3′	5′-GGCACTTGGAGGAATAAACTG-3′
PEPT1	5′-TGTCCACCGCCATCTACCATA-3′	5′-CCACGAGTCGGCGATAAGAG -3′
LAT2	5′-AGGCTGGAACTTTCTGAATTACG-3′	5′-ACATAAGCGACATTGGCAAAGA-3′
HIF1a	5′-ATCCATGTGACCATGAGGAAATG-3′	5′-TCGGCTAGTTAGGGTACACTTC-3′
IL4	5′-AGTGTCCTTCTCATGGTGGC-3′	5′-CACCGAGTTGACCGTAACAG-3′
IL17	5′-CACTTTGCCTCCCAGATCAC-3′	5′-ACCAATCCCAAAAGGTCCTC-3′
IFNϒ	5′-TGGCTTTTCAGCTCTGCATC-3′	5′-CCGCTACATCTGAATGACCTG-3′
TNFα	5′-TCAATCGGCCCGACTATCTC-3′	5′-CAGGGCAATGATCCCAAAGT-3′
IL10	5′-TCCCTGTGAAAACAAGAGCA-3′	5′-ATAGAGTCGCCACCCTGATG-3′
Foxp3	5′- CACCTGGCTGGGAAAATGG-3′	5′-GGAGCCCTTGTCGGATGAT-3′

**Table 2:** Culture medium and antibiotic used as growth inhibition control for included bacterial strains.

Strain	Culture Medium	Antibiotics Used as Growth Inhibition Control
*Escherichia coli* ATCC 25922	BHI	colistin (2 μg/mL)
*Straphylococcus aureus* ATCC 29213	BHI	ampicillin (50 μg/mL)
*Lactobacillus rhamnosis*	BHI	erythromycin (10 μg/mL)
*Bifidobacterium longum*	MRS (+0.05% cystein)	erythromycin (10 μg/mL)

Abbreviations: ATCC—American Type Culture Collection; BHI—brain heart infusion; MRS—De Man, Rogosa and Sharpe agar.

**Table 3:** Composition of interventions.

Product	WPI	DH28	DH51
Protein (%)	90.0	86.5	85.1
Lactose (%)	0.05	0.10	0.09
Fat (%)	0.10	0.10	0.07
Ash (%)	4.0	3.3	4.9
Mn (Da)	N/A	593	333
Mw (Da)	N/A	914	581
<375 Da (%)		16.1	37.3
375–750 Da (%)		35.9	36.4
750–1250 Da (%)		24.6	19.4
1250–2500 Da (%)		21.4	6.6
>2500 Da (%)		2.1	0.3
DH (%)		27.7	50.9
FAA (%)	0.0	0.5	29.0

Abbreviations: Mn—number average molecular weight; Mw—weight average molecular weight; DH—degree of hydrolysis; FAA—free amino acids; N/A—not applicable.

## Data Availability

Data is contained within the article or supplementary material.

## Supplementary Materials

The following supporting information can be downloaded at: https://www.mdpi.com/article/10.3390/nu15020393/s1, Figure S1. Apical—basolateral orientation of human intestinal epithelial cells in the HIO monolayer model; Figure S2. Experimental set-up of the experiment assessing HIF1A mRNA expression over time during hypoxia and additional 3D paracellular barrier experiments for HIO model development; Figure S3. mRNA expression of HIF1A over time during hypoxia (2 h, 6 h, 14 h and 24 h of hypoxia) compared to a normoxic control in crypt-like HIO monolayers; Figure S4. Measurement of the number and CD25 and CD69 expression of CD4+CD25^high^CD127^low^ Treg in non-activated PBMCs and proliferation; Figure S5. Measurement of the proliferation of CD4+ and CD8+ T cells in T cell-activated PBMCs; Table S1. Composition of interventions.
