# Supplementary material for: Hypoxia-Driven Changes in a Human Intestinal Organoid Model and the Protective Effects of Hydrolyzed Whey
Source: Nutrients. 2023 Jan 12;15(2):393. doi: 10.3390/nu15020393 (PMC9863820; doi:10.3390/nu15020393)
Supplement: Supplementary file 1 [file nutrients-15-00393-s001.zip › nutrients-2128194-supplementary.pdf]

nutrients

Supplementary Materials

Hypoxia-Driven Changes in a Human Intestinal Organoid Model and the Protective Effects of Hydrolyzed Whey

Ilse H. de Lange, Charlotte van Gorp, Kimberly R.I. Massy, Lilian Kessels, Nico Kloosterboer, Ann Bjørnshave, Marie Stampe Ostenfeld, Jan G.M.C. Damoiseaux, Joep P.M. Derikx, Wim G. van Gemert and Tim G.A.M. Wolfs *

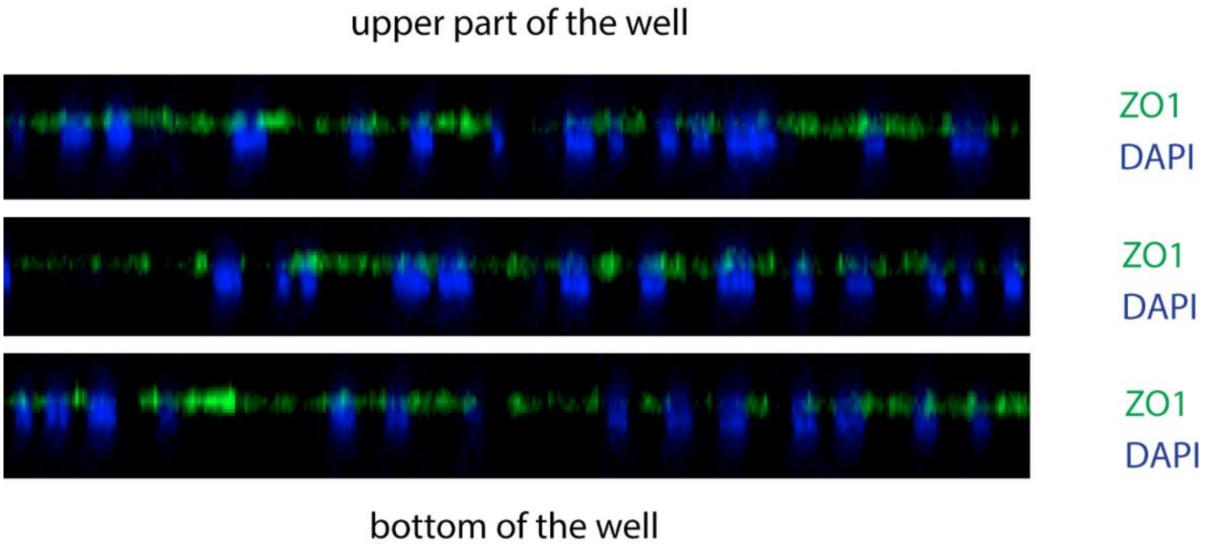

Supplementary Figure S1. Apical–basolateral orientation of human intestinal epithelial cells. in the HIO monolayer model with the apical side facing the upper part of the well was detected through localization of ZO1 (green) relative to the cell nucleus (DAPI, blue).

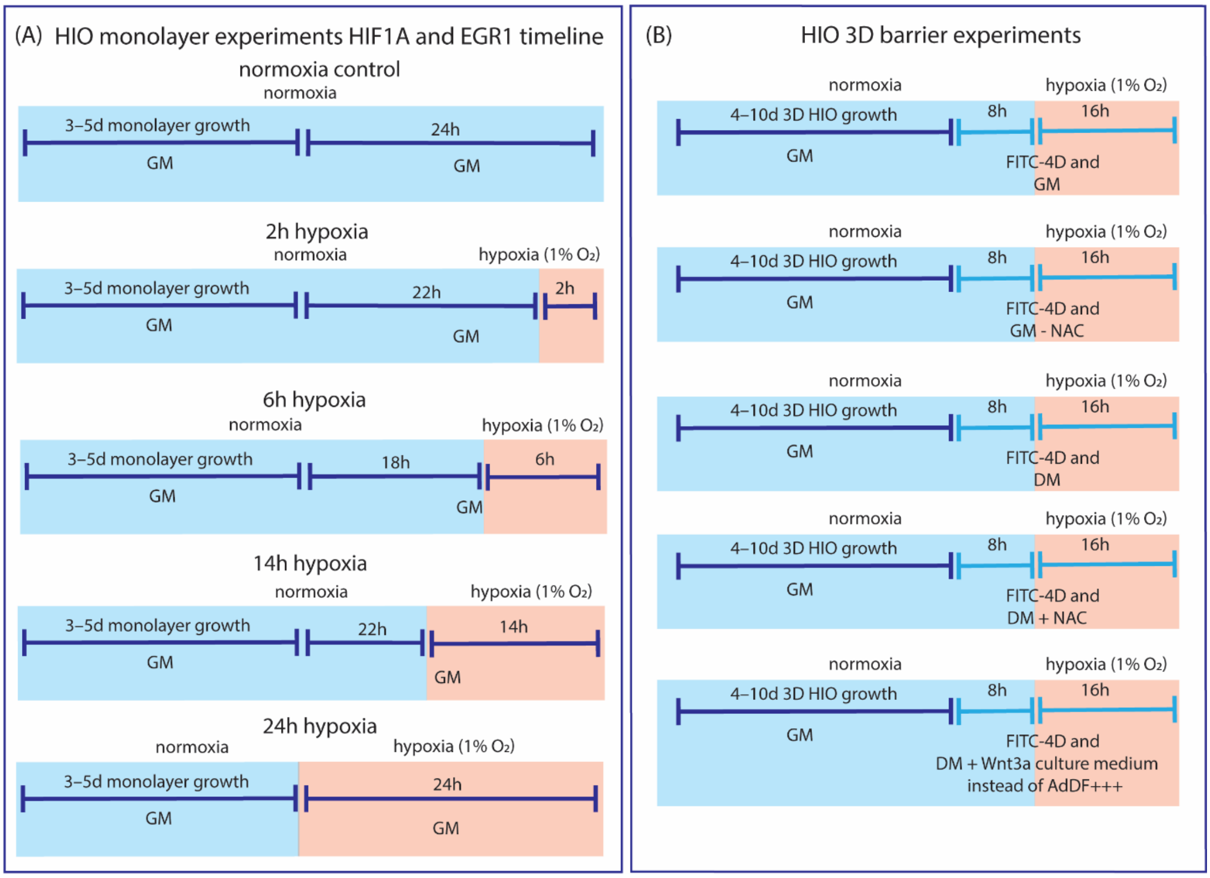

Supplementary Figure S2. Experimental set-up of the experiment assessing HIF1A mRNA expression over time during hypoxia and additional 3D paracellular barrier experiments for HIO model development. (**A**) For assessing HIF1A mRNA expression over time during hypoxia in crypt-like HIO monolayers, HIO monolayers were cultured for 3–5d prior to the onset of the experiment. Subsequently, organoids were cultured with GM for an additional 24h and exposed to 0h, 2h, 6h, 14h or 24h of hypoxia (1% O_2) before end-of-experiment. (**B**) For additional paracellular barrier experiments with 3D crypt-like and villus-like HIO, HIO were cultured with GM for 4–10d prior to the onset of the experiment. 24h before analyses, FITC-D4 was added to the GM or DM with or without supplemented NAC. In addition, in one group DM was used in which the surplus of AdDF+++ was replaced by the DMEM medium used for the production of Wnt3a conditioned medium. All groups were exposed to 16h of hypoxia (1% O_2) prior to analysis.

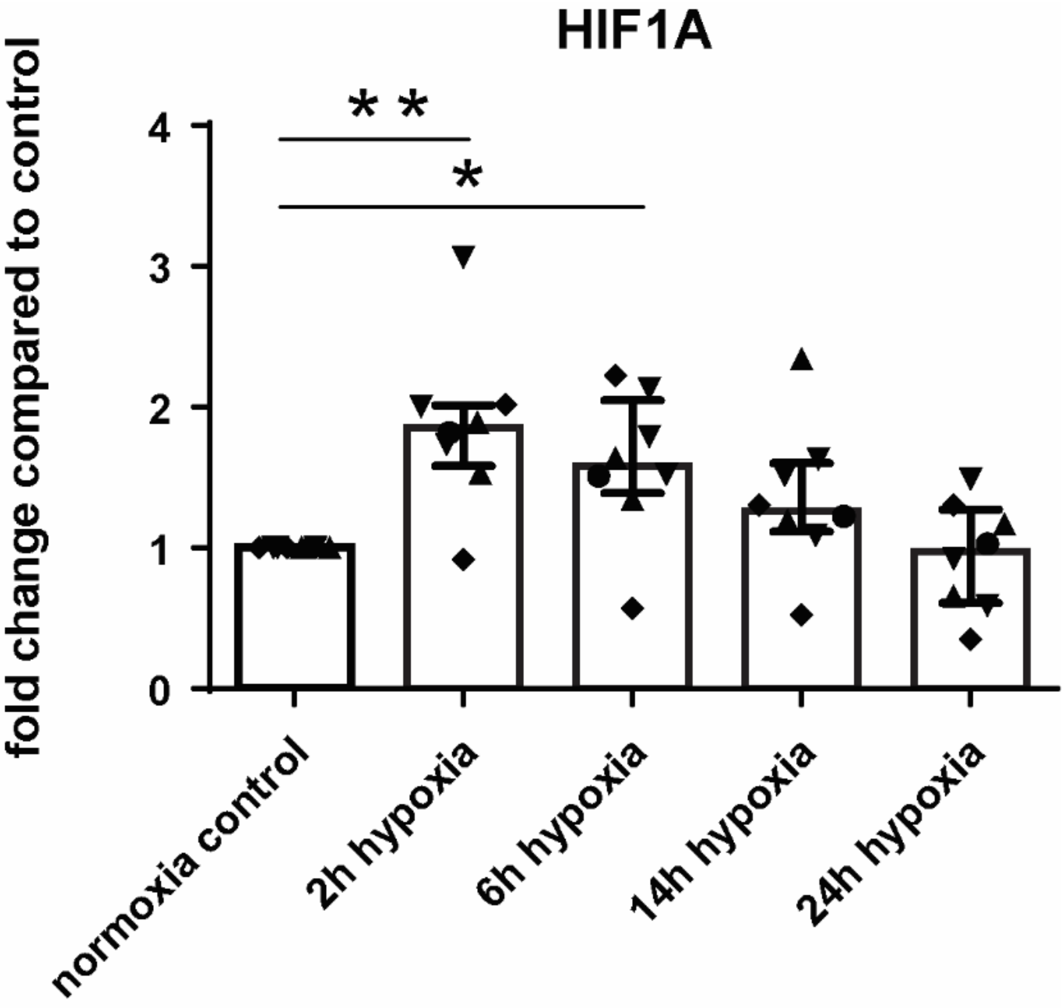

Supplementary Figure S3. mRNA expression of HIF1A over time during hypoxia (2h, 6h, 14h and 24h of hypoxia) compared to a normoxic control in crypt-like HIO monolayers. Data are reported as relative expression compared to control (set at 1) and displayed as median with interquartile range. Results were obtained from 4 different HIO donors (depicted by different data point symbols, one symbol type per donor). *$p \leq 0.05$, **$p \leq 0.01$.

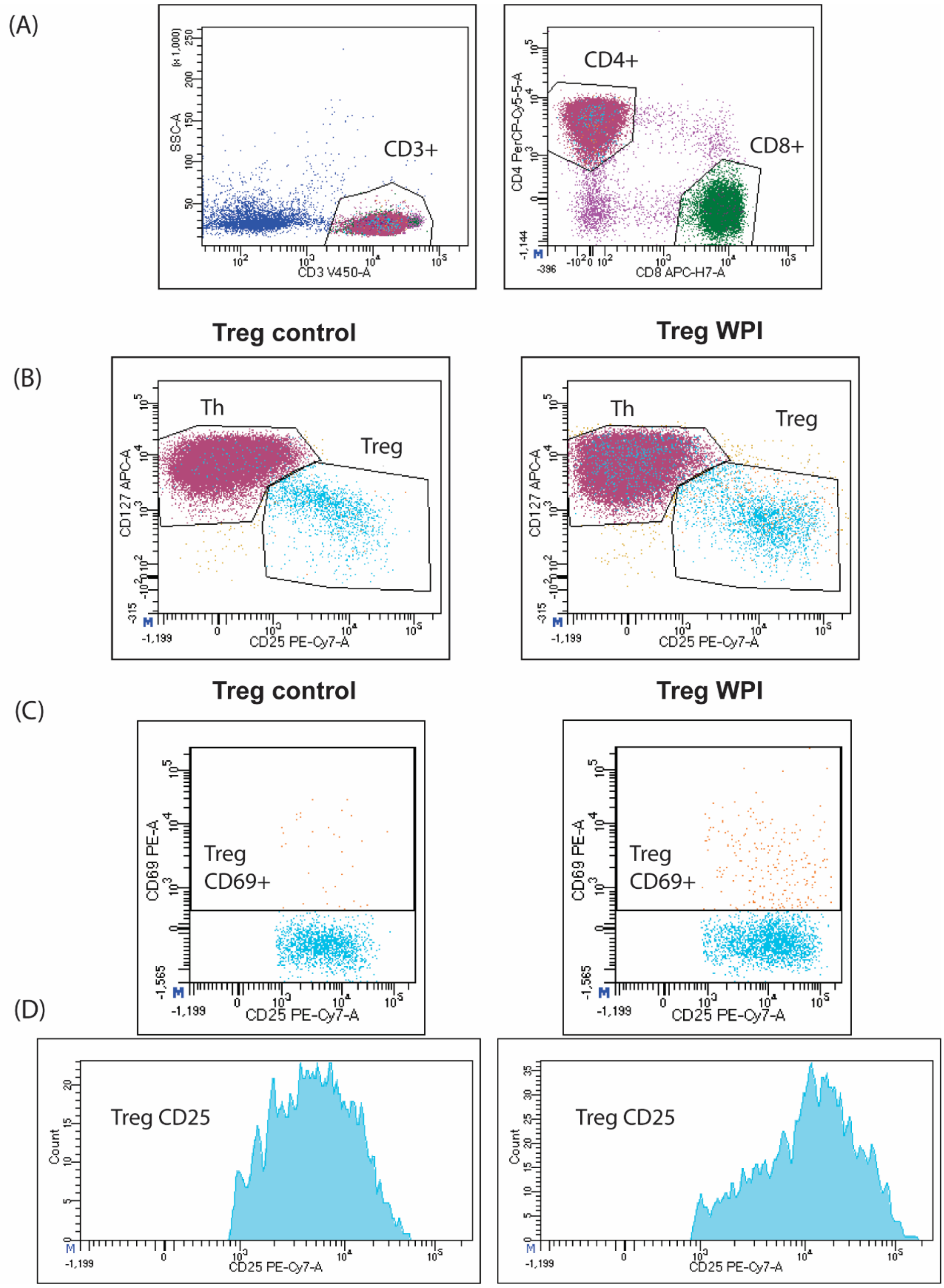

Supplementary Figure S4. Measurement of the number and CD25 and CD69 expression of CD4+CD25^{high}CD127^{low} Treg in non-activated PBMCs and proliferation (**A**) Single cells were selected from PBMCs in forward and side scatter and live cells were selected with a live-dead marker. From this population, the CD3+ cells were picked and the amount of CD4+ and CD8+ cells was determined. Within the population of CD4+ T cells, CD25 and CD127 expression was determined and the percentage of CD4+CD25^{high}CD127^{low} Treg was measured. (**B**) Relative to controls, incubation with WPI increased the percentage of CD4+ T cells that is Treg. (**C**) Relative to controls, incubation with WPI

increased the % of Treg that is CD69+. (**D**) Relative to controls, incubation with WPI increased the CD25 expression of Treg (CD25 MFI). Abbreviations: CD3, cluster of differentiation 3; CD4, cluster of differentiation 4; CD8, cluster of differentiation 8; WPI, whey protein isolate; MFI, mean fluorescent intensity.

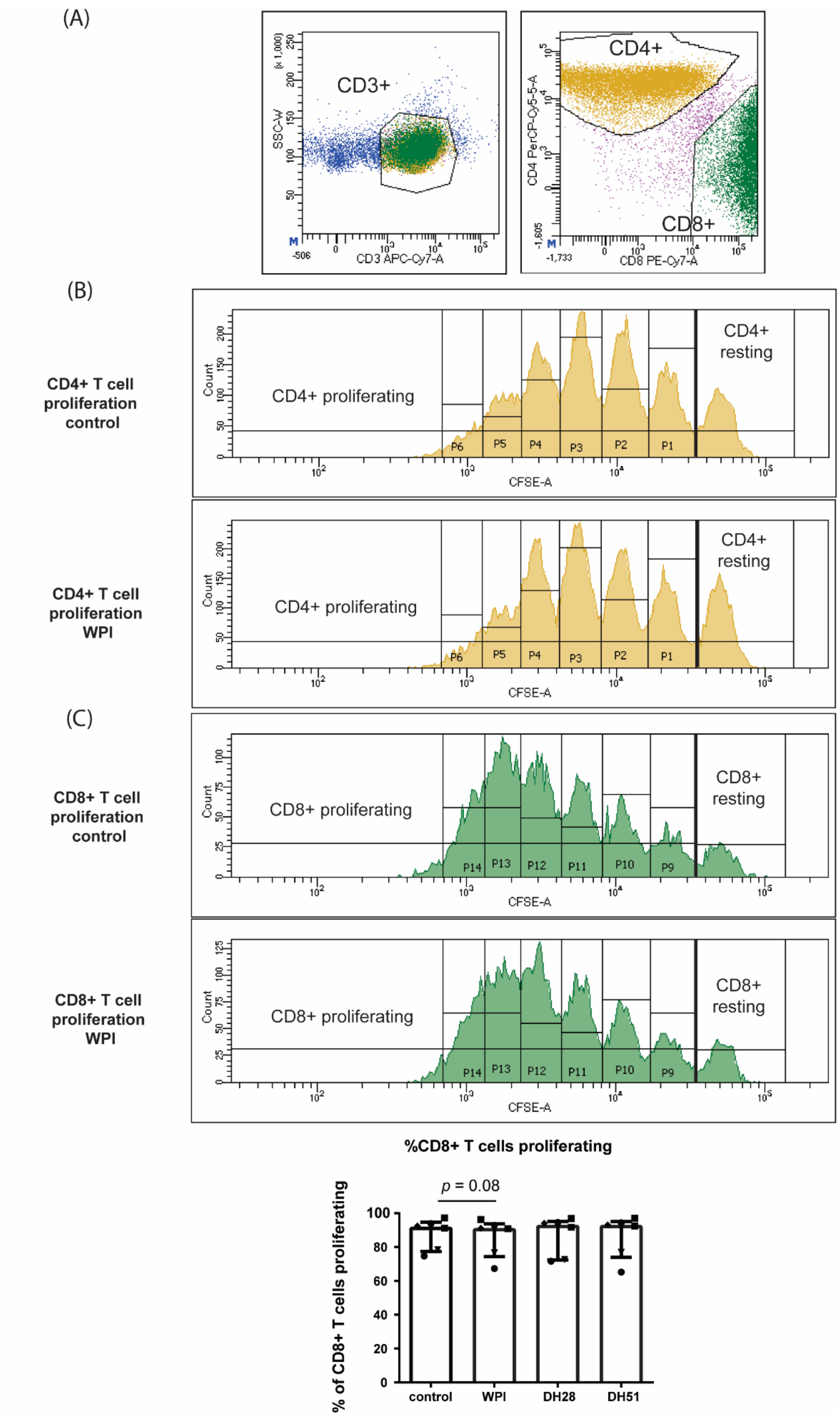

Supplementary Figure S5. Measurement of the proliferation of CD4+ and CD8+ T cells in T-cell activated PBMCs. (**A**) Single cells were selected from PBMCs in forward and side scatter and live cells were selected with a live-dead marker. From this population, the CD3+ cells were picked and the amount of CD4+ and

CD8+ cells was determined. (**B**) Within the population of CD4+ T cells the percentage of proliferating cells was determined with a CFSE proliferation assay. Incubation with WPI for 5 days decreased CD4+ T cell proliferation. **(C**) Within the population of CD8+ T cells the percentage of proliferating cells was determined with a CFSE proliferation assay. Incubation with WPI for 5 days tended to decrease CD8+ T cell proliferation. Results were obtained from four different PBMC donors (depicted by different data point symbols). Abbreviations: CD3, cluster of differentiation 3; CD4, cluster of differentiation 4; CD8, cluster of differentiation 8; WPI, whey protein isolate.

Supplementary Table S1. Composition of interventions.

**FAA distribution (mg/100g powder)**	**WPI**	**DH28**	**DH51**
Alanine		44.3	1250
Arginine		<10	1050
Asparagine		17.2	1650
Aspartic acid		12.2	1020
Cystine		<10	179
Glutamine		<10	1050
Glutamic acid		69.4	1050
Glycine		<10	113
Histidine		11.9	748
Isoleucine		15.3	2370
Leucine		102	5420
Lysine		53.5	3990
Methionine		11.3	1200
Phenylalanine		57.3	1660
Proline		<10	35.9
Serine		28.9	1160
Threonine		33.4	1600
Tryptophane		20.2	410
Tyrosine		29.6	993
Valine		34.9	2110
**AA (g/100 g AA)**	**WPI**	**DH28**	**DH51**
Alanine	4.91	5.39	5.43
Arginine	1.88	1.75	1.78
Asparagine+Aspartic acid	10.99	11.44	11.29
Cystine	2.4	2	2.15
Glutamine+Glutamic acid	16.9	18.93	18.41
Glycine	1.58	1.53	1.51
Histidine	1.52	1.53	1.57
Isoleucine	6.43	6.28	6.84
Leucine	10.08	9.6	9.60
Lysine	9.28	9.71	9.83
Methionine	2.13	2.22	2.08
Phenylalanine	2.81	2.36	2.39
Proline	6.56	5.98	5.62
Serine	4.89	4.74	4.74
Threonine	7.34	7.38	7.35
Tryptophane	1.85	1.27	1.24
Tyrosine	2.74	2.3	2.31
Valine	5.71	5.61	5.87

Abbreviations: AA, amino acids, FAA, free amino acids, WPI, whey protein isolate; DH28, whey protein hydrolysate with 27.7% degree of hydrolysis; DH51, whey protein hydrolysate with 50.9% degree of hydrolysis.
